# Supplementary material for: Anti-TNF (adalimumab) injection for the treatment of pain-predominant early-stage frozen shoulder: the Anti-Freaze-Feasibility randomised controlled trial
Source: BMJ Open. 2024 May 1;14(5):e078273. doi: 10.1136/bmjopen-2023-078273 (PMC11086567; doi:10.1136/bmjopen-2023-078273)
Supplement: Supplementary data [file bmjopen-2023-078273supp001.pdf]

Supplementary materials (online only)

Figure S1: SPADI score: change from baseline and at 3 months

a) Total SPADI Scores at baseline and 3 months for each participant

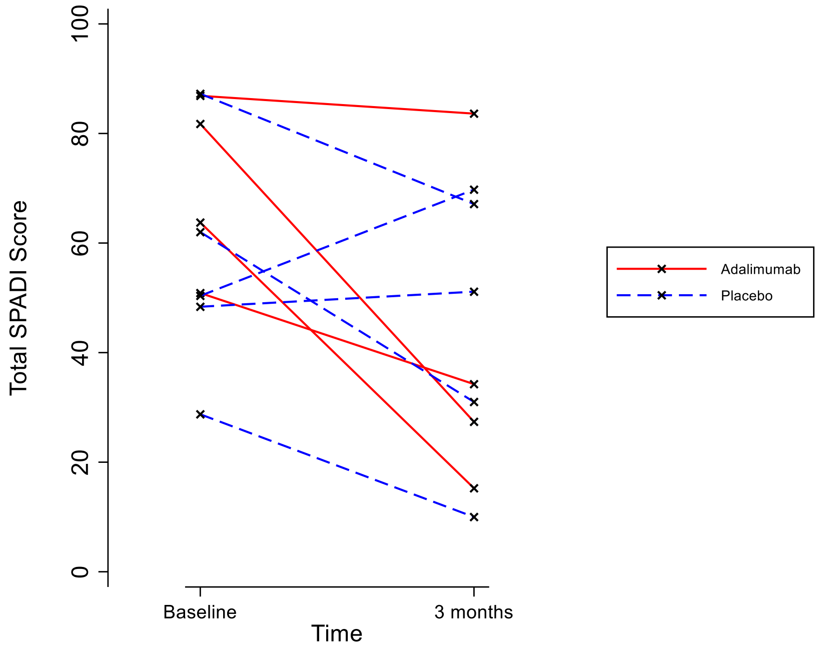

b) SPADI Pain Subscale Scores at baseline and 3 months for each participant

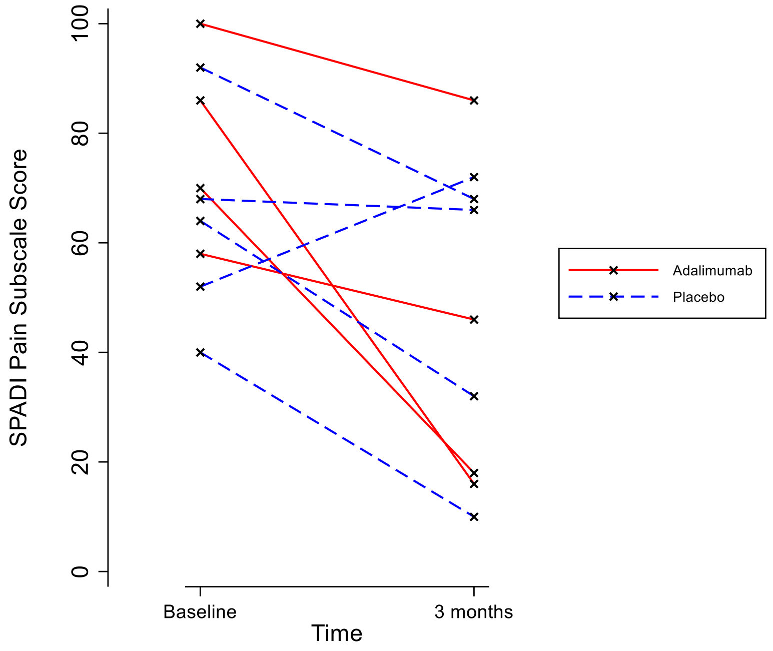

Hopewell S, *et al. BMJ Open* 2024; 14:e078273. doi: 10.1136/bmjopen-2023-078273

c) SPADI Function Subscale Scores at baseline and 3 months for each participant

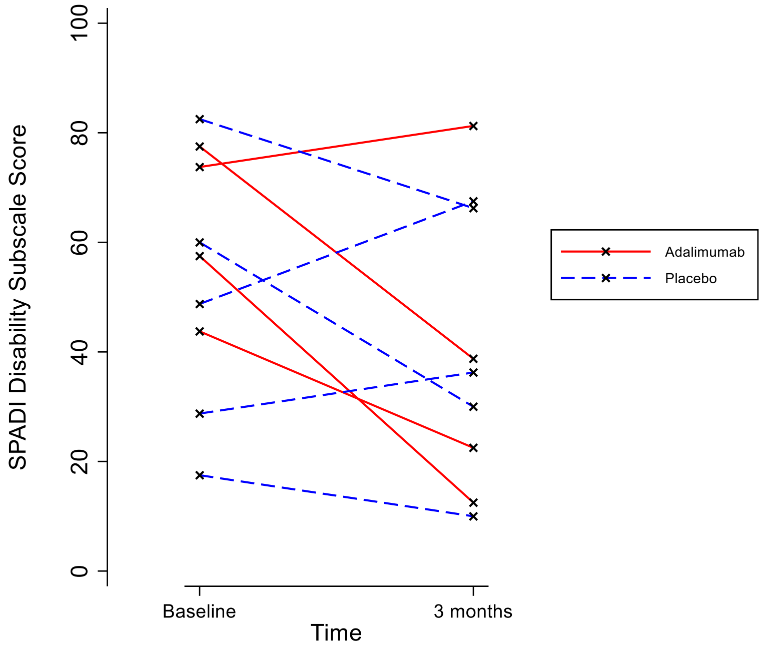

Hopewell S, *et al. BMJ Open* 2024; 14:e078273. doi: 10.1136/bmjopen-2023-078273

Table S1: Secondary patient-reported outcomes

		Adalimumab[1] (n=4)	Placebo[1] (n=5)
SPADI Total score	Baseline	70.8 (16.6)	55.4 (21.5)
	3 months	40.1 (30.0)	45.8 (25.3)
SPADI pain (subscale)	Baseline	78.5 (18.4)	63.2 (19.5)
	3 months	41.5 (32.7)	49.6 (27.3)
SPADI disability (subscale)	Baseline	63.1 (15.6)	47.5 (25.7)
	3 months	38.8 (30.3)	42 (24.7)
FAB-Q	Baseline	17.3 (3.3)	17.4 (3.6)
	3 months	9.3 (6.2)	12.8 (5.9)
PSEQ – short-form	Baseline	9 (3.6)	9.2 (1.9)
	3 months	10.5 (1.3)	8.2 (2.8)
ISI	Baseline	17.8 (5.)	17.6 (4.0)
	3 months	9.8 (6.7)	13.2 (7.6)
Return to desired activities	Baseline	9 (4.5)	9.4 (2.7)
	3 months	5.5 (3.8)	8.4 (3.6)
Global impression of change	3 months	2.3 (2.2)	0 (3.0)

[1] *Summaries are mean (SD)*

SPADI: Shoulder Pain And Disability Index; FAB-Q: Fear-Avoidance Belief Questionnaire; PSEQ: Pain Self-Efficacy Questionnaire; ISI: Insomnia Severity Index

Hopewell S, *et al. BMJ Open* 2024; 14:e078273. doi: 10.1136/bmjopen-2023-078273

Table S2: Clinician-assessed range of shoulder motion

	Adalimumab[1] (n=4)	Placebo[1] (n=5)
**Flexion**		
Baseline	71.3 (26.3)	101.6 (24.7)
3 months	112.5 (54.8)	140 (25.7)
**Extension**		
Baseline	26.3 (18.0)	27.6 (9.5)
3 months	30.0 (8.2)	40.6 (9.8)
**Abduction**		
Baseline	50.0 (30.3)	69.0 (26.6)
3 months	105.0 (56.1)	115.0 (42.7)
**Internal rotation**		
Baseline	23.8 (18.9)	40.2 (40.8)
3 months	21.3 (17.0)	41 (37.8)
**External rotation**		
Baseline	22.5 (8.7)	15.0 (16.9)
3 months	28.8 (6.3)	55.0 (16.2)

[1] *Summaries are mean (SD) degrees of shoulder motion*

Table S3: Patient reported range of shoulder motion

	Adalimumab[1] (n=4)	Placebo[1] (n=5)
**Flexion**		
Baseline	102.3 (21.3)	112.4 (21.3)
3 months	117.3 (38.4)	145.6 (22.9)
**Extension**		
Baseline	26.3 (6.9)	23.7 (8.5)
3 months	23.5 (7.0)	36.5 (7.0)
**Abduction**		
Baseline	81.3 (35.4)	99.3 (27.0)
3 months	79.3 (45.7)	134.2 (52.5)
**Internal rotation**		
Baseline	18.5 (19.8)	29 (29.9)
3 months	51.0 (15.6)	72.3 (19.4)
**External rotation**		
Baseline	35.3 (15.7)	24.8 (14.5)
3 months	35.5 (9.8)	37.0 (24.0)

[1] *Summaries are mean (SD) degrees of shoulder motion*

Hopewell S, *et al. BMJ Open* 2024; 14:e078273. doi: 10.1136/bmjopen-2023-078273
