# Supplementary material for: Anti-TNF (adalimumab) injection for the treatment of pain-predominant early-stage frozen shoulder: the Anti-Freaze-Feasibility randomised controlled trial
Source: BMJ Open. 2024 May 1;14(5):e078273. doi: 10.1136/bmjopen-2023-078273 (PMC11086567; doi:10.1136/bmjopen-2023-078273)
Supplement: Author's manuscript [file bmjopen-2023-078273.draft_revisions.pdf]

BMJ Open is committed to open peer review. As part of this commitment we make the peer review history of every article we publish publicly available.

When an article is published we post the peer reviewers' comments and the authors' responses online. We also post the versions of the paper that were used during peer review. These are the versions that the peer review comments apply to.

The versions of the paper that follow are the versions that were submitted during the peer review process. They are not the versions of record or the final published versions. They should not be cited or distributed as the published version of this manuscript.

BMJ Open is an open access journal and the full, final, typeset and author-corrected version of record of the manuscript is available on our site with no access controls, subscription charges or pay-per-view fees (http://bmjopen.bmj.com).

If you have any questions on BMJ Open's open peer review process please email info.bmjopen@bmj.com

BMJ Open

Anti-TNF (adalimumab) injection for the treatment of pain predominant early-stage frozen shoulder: the Anti-Freaze-Feasibility randomised controlled trial.

Journal:	*BMJ Open*
Manuscript ID	bmjopen-2023-078273
Article Type:	Original research
Date Submitted by the Author:	31-Jul-2023
Complete List of Authors:	Hopewell, Sally; University of Oxford, Oxford Clinical Trials Research Unit, Nuffield Department of Orthopaedics, Rheumatology and Musculoskeletal Sciences<br>Srikesavan, Cynthia; University of Oxford, Centre for Rehabilitation Research in Oxford, Nuffield Department of Orthopaedics, Rheumatology and Musculoskeletal Sciences; University of Exeter, College of Medicine and Health<br>Evans, Alison; University of Oxford, Kennedy Institute of Rheumatology, Nuffield Department of Orthopaedics, Rheumatology and Musculoskeletal Sciences<br>Er, Fema; University of Oxford, Oxford Clinical Trials Research Unit, Nuffield Department of Orthopaedics, Rheumatology and Musculoskeletal Sciences<br>Rangan, Amar; The James Cook University Hospital, South Tees Hospitals NHS Foundation Trust; University of York, Department of Health Sciences<br>Preece, Jane; Patient and Public Involvement Representative<br>Francis, Anne; University of Oxford, Oxford Clinical Trials Research Unit, Nuffield Department of Orthopaedics, Rheumatology and Musculoskeletal Science<br>Massa, M Sofia; University of Oxford, Oxford Clinical Trials Research Unit, Nuffield Department of Orthopaedics, Rheumatology and Musculoskeletal Sciences<br>Feldmann, Marc; University of Oxford, Kennedy Institute of Rheumatology, Nuffield Department of Orthopaedics, Rheumatology and Musculoskeletal Sciences<br>Lamb, Sarah; University of Exeter, College of Medicine and Health<br>Nanchahal, Jagdeep; The University of Oxford, Kennedy Institute of Rheumatology, Nuffield Department of Orthopaedics, Rheumatology and Musculoskeletal Sciences
Keywords:	Patients, Shoulder < ORTHOPAEDIC & TRAUMA SURGERY, Musculoskeletal disorders < ORTHOPAEDIC & TRAUMA SURGERY

SCHOLARONE™
Manuscripts

I, the Submitting Author has the right to grant and does grant on behalf of all authors of the Work (as defined in the below author licence), an exclusive licence and/or a non-exclusive licence for contributions from authors who are: i) UK Crown employees; ii) where BMJ has agreed a CC-BY licence shall apply, and/or iii) in accordance with the terms applicable for US Federal Government officers or employees acting as part of their official duties; on a worldwide, perpetual, irrevocable, royalty-free basis to BMJ Publishing Group Ltd ("BMJ") its licensees and where the relevant Journal is co-owned by BMJ to the co-owners of the Journal, to publish the Work in this journal and any other BMJ products and to exploit all rights, as set out in our licence.

The Submitting Author accepts and understands that any supply made under these terms is made by BMJ to the Submitting Author unless you are acting as an employee on behalf of your employer or a postgraduate student of an affiliated institution which is paying any applicable article publishing charge ("APC") for Open Access articles. Where the Submitting Author wishes to make the Work available on an Open Access basis (and intends to pay the relevant APC), the terms of reuse of such Open Access shall be governed by a Creative Commons licence – details of these licences and which Creative Commons licence will apply to this Work are set out in our licence referred to above.

Other than as permitted in any relevant BMJ Author's Self Archiving Policies, I confirm this Work has not been accepted for publication elsewhere, is not being considered for publication elsewhere and does not duplicate material already published. I confirm all authors consent to publication of this Work and authorise the granting of this licence.

For peer review only

Anti-TNF (adalimumab) injection for the treatment of pain predominant early-stage frozen shoulder: the Anti-Freaze-Feasibility randomised controlled trial.

Sally Hopewell[1], Cynthia Srikesavan[2,3], Alison Evans[4], Fema Er[1], Amar Rangan[5,6], Jane Preece[7], Anne Francis[1], Sofia Massa[1], Marc Feldmann[3], Sarah E Lamb[5], Jagdeep Nanchahal[4]

1. Oxford Clinical Trials Research Unit, Nuffield Department of Orthopaedics, Rheumatology and Musculoskeletal Sciences, University of Oxford, UK.
2. Centre for Rehabilitation Research in Oxford, Nuffield Department of Orthopaedics, Rheumatology and Musculoskeletal Sciences, University of Oxford, UK
3. College of Medicine and Health, University of Exeter, UK.
4. Kennedy Institute of Rheumatology, Nuffield Department of Orthopaedics, Rheumatology and Musculoskeletal Sciences, University of Oxford, UK.
5. James Cook University Hospital, South Tees Hospitals NHS Foundation Trust, Middlesbrough, UK.
6. Health Sciences and HYMS, University of York, UK
7. Patient and Public Involvement Representative, Kidderminster, UK

Correspondence to: Professor Sally Hopewell, Oxford Clinical Trials Research Unit / Centre for Statistics in Medicine, Nuffield Department of Orthopaedics, Rheumatology and Musculoskeletal Sciences, University of Oxford. Email: sally.hopewell@csm.ox.ac.uk

For peer review only

ABSTRACT

Objective: The Anti-Freaze-F (AFF) trial assessed the feasibility of conducting a definitive trial to determine whether intra-articular injection of adalimumab can reduce pain and improve function in people with pain-predominant early-stage frozen shoulder.

Design: Multi-centre, randomised feasibility study, with embedded qualitative study.

Setting: Four UK NHS musculoskeletal and related physiotherapy services.

Participants: Adults ≥18 years with new episode of shoulder pain attributable to early-stage frozen shoulder.

Interventions: Participants were randomised (centralised computer generated 1:1 allocation) to either ultrasound guided intra-articular injection of: 1) adalimumab (160mg) or 2) placebo (saline [0.9% sodium chloride]). Participants were blinded to treatment allocation. Second injection of allocated treatment (adalimumab 80mg) or equivalent placebo was administered 2-3 weeks later.

Primary feasibility objectives: 1) ability to screen and identify participants; 2) willingness of eligible participants to consent and be randomised; 3) practicalities of delivering the intervention; 4) standard deviation of the Shoulder Pain and Disability Index (SPADI) score and attrition rate at 3 months.

Results: Between 31 May 2022 and 7 February 2023, 156 patients were screened of whom 39 (25%) were eligible. The main reasons for ineligibility were other shoulder disorder (38%; n=45/117) or no longer in pain-predominant frozen shoulder (33%; n=39/117). Of the 39 eligible patients, nine (23%) consented to be randomised (adalimumab n=4; placebo n=5). The main reason patients declined was because they had a preference for receiving steroid injection (n=13). All participants received treatment as allocated. The mean time from randomisation to first injection was 12.3 (adalimumab) and 7.2 days (placebo). Completion rates for patient reported and clinician assessed outcomes were 100%.

Conclusion: This study demonstrated that current NHS musculoskeletal physiotherapy settings yielded only small numbers of participants, too few to make a trial viable. This was because many patients had passed the early-stage of frozen shoulder or had already formulated a preference for treatment.

Trial registration: ISRCTN 27075727, EudraCT 2021-03509-23, ClinicalTrials.gov NCT05299242. (REC 21/NE/0214).

For peer review only - http://bmjopen.bmj.com/site/about/guidelines.xhtml

STRENGHTENS AND LIMITATIONS OF THIS STUDY

- To our knowledge, AFF is the first study to assess the feasibility of conducting a large multi-centre randomised trial to test the effectiveness of a new treatment for people with early stage, pain-predominant frozen shoulder. This is important given the evidence for the limited effectiveness of current treatments.
- Despite not meeting our feasible objectives in terms of recruitment, it was feasible to deliver intra-articular injection of adalimumab in line with the trial protocol and all participants completed the 3-month follow up assessment.
- One of the main reasons people were not eligible to take part in this study was because they were no longer in the pain-predominant phase of frozen shoulder by the time they were seen by a physiotherapist.
- Another major barrier to feasibility of the trial was because some people, who were eligible and approached to take part, already had a treatment preference for receiving a steroid injection.
- Our embedded qualitative study provides a useful insight into patient experiences with the early painful phase of frozen shoulder and their perceptions about being involved in the AFF trial.

For peer review only

INTRODUCTION

Frozen shoulder (adhesive capsulitis) is a common and extremely painful and debilitating condition. Affected individuals struggle with activities of daily living and significant sleep disturbances as a result of severe pain [1]. It affects about 9% of people in the United Kingdom (UK) aged 25-64 years [2], and 20% develop the same problem in the other shoulder [3]. Frozen shoulder may develop as a primary condition or secondarily following surgery or trauma.

The classic description of the development of frozen shoulder is of three overlapping phases [4, 5]. The initial pain-predominant inflammatory phase is characterised by constant pain and difficulty sleeping and lasts between 3 and 9 months. This progresses to a stiffness-predominant fibrotic phase, with progressive restriction of motion, particularly external rotation and elevation of the shoulder, and impairment of function, and lasts between 4 and 12 months. The pain changes from being constant to being manifest at the end of range of motion and of reduced intensity. Over the final phase, there is a gradual improvement in range of motion and stiffness over a 12 to 48-month period, although pain may persist with end-range movements. The average duration of the condition is 30 months (range 1 to 3.5 years) although full resolution of symptoms does not always occur [6].

The aetiology of frozen shoulder is poorly understood and consequently there is no consensus on the optimal treatment. The majority of patients with early-stage pain predominant frozen shoulder are managed in primary care or at primary care interface musculoskeletal services by physiotherapists and general practitioners. During this stage, standard treatment consists of rest, advice, analgesics, physiotherapy and corticosteroid injections to address the symptoms. Current treatments available to patients with frozen shoulder are of limited efficacy. Two Cochrane reviews concluded that whilst oral steroid or local steroid injections lead to short-term benefit in pain and range of motion, the effects are not maintained beyond 6 weeks [7-9]. Other Cochrane reviews concluded that there is no evidence that physiotherapy or ultrasound therapy are beneficial [10], and that manual therapy with exercise is less effective than corticosteroid injection in the short-term [11]. The UK FROST compared the effects of physiotherapy plus corticosteroid injection, manipulation under anaesthesia with a steroid injection, and arthroscopic capsular release supplemented with manipulation [9]. None of the treatments were found to be clinically superior.

Our study (Anti-Freaze-F) was designed to specifically target people with early-stage pain predominant frozen shoulder. We assessed the feasibility of conducting a large multicentre randomised trial to test whether giving an intra-articular injection of adalimumab (a drug targeting the inflammatory mediator

For peer review only - http://bmjopen.bmj.com/site/about/guidelines.xhtml

tumour necrosis factor [TNF]), can reduce pain and prevent the condition from getting worse, if given during the early pain predominant stage. Whilst the pathogenesis of frozen shoulder remains largely unknown, a recent systematic review confirmed the presence of fibrosis and the role of inflammation [6]. The affected tissues are infiltrated by immune cells and there are elevated levels of pro-inflammatory cytokines, including tumour necrosis factor (TNF) [12, 13], with myofibroblasts driving the development of fibrosis [14]. Approximately 50% of people with frozen shoulder also develop Dupuytren's disease [15] and the underlying pathology of frozen shoulder has similarities to Dupuytren's disease [14, 16] In a dose-ranging proof of concept phase 2a followed by a 2b clinical trial (RIDD trial) we found that injection of anti-TNF (adalimumab) directly into Dupuytren's nodules led to significant downregulation of the myofibroblast phenotype and reduction in nodule hardness (primary endpoint) and nodule size on ultrasound scan (secondary endpoint)[17, 18].

Objectives

The aim of the Anti-Freaze-F (AFF) trial was to assess the feasibility of conducting a large randomised controlled trial to assess whether an intra-articular injection of adalimumab (anti-TNF) can reduce pain and improve function in people with early-stage frozen shoulder. The primary objectives were to assess the:

- ability to screen and identify potential participants with pain predominant early stage frozen shoulder;
- willingness of eligible participants to consent and be randomised to intervention;
- practicalities of delivering the intervention, including time to first injection and number of participants receiving second injection;
- standard deviation (SD) of the Shoulder Pain and Disability Index (SPADI) score and attrition rate at 3 months (i.e. 12 weeks) post-randomisation in order to estimate the sample size for a definitive trial.

Secondary objectives were to assess the follow up rates and viability of patient reported outcome measures and clinician assessed range of shoulder motion at 3 months (i.e. 12 weeks).

The objectives of the embedded qualitative study were to explore the participant experience of being recruited to the AFF trial, the treatment received and follow-up schedule, and to understand what helps participant recruitment to the trial intervention.

METHODS

Study design

For peer review only - http://bmjopen.bmj.com/site/about/guidelines.xhtml

A multi-centre, randomised, double-blind, parallel group, feasibility trial with an embedded qualitative study.

Setting

Participants were recruited from four NHS musculoskeletal services and their related physiotherapy services, with treatment delivered within these services or local secondary care dependant on the local service provision.

Study participants

Participants were eligible if they were aged 18 years and above with a new episode of shoulder pain attributable to pain-predominant stage of frozen shoulder (i.e. within approximately three to nine months of onset of symptoms) diagnosed using criteria set out in the British Elbow and Shoulder Society (BESS) guidelines [19]. Patients were excluded if they had frozen shoulder secondary to significant shoulder trauma; other shoulder disorders (e.g., inflammatory arthritis, rotator cuff disorders, glenohumeral joint instability) or with red flags consistent with the criteria set out in the BESS guidelines [19]; bilateral early stage frozen shoulder; had received corticosteroid injection for shoulder pain in the last 12 weeks; were currently taking any anti-TNF drug, or being treated with coumarin anticoagulants, such as warfarin; had significant renal or hepatic impairment, or had contra-indications to anti-TNF injection. Detailed inclusion and exclusion criteria are described in the trial protocol [20]. Patients who met the eligibility criteria and wanted to participate were approached for written informed consent by a research facilitator trained in Good Clinical Practice at each participating site. Ethics approval was obtained from the National Research Ethics Committee and MHRA (REC 21/NE/0214).

Randomisation

Consented participants were randomly assigned to receive either: 1) intra-articular injection of adalimumab; or 2) placebo injection (saline [0.9% sodium chloride], both under ultrasound guidance. Randomisation (1:1) was done by the research facilitator at each site using the centralised randomisation service provided by the Oxford Clinical Trials Research Unit once the patient was enrolled and baseline assessment and questionnaire completed. Randomisation was computer-generated and stratified by study site using a variable block size to ensure the participants from each study site had equal chance of receiving either intervention.

Blinding

For peer review only - http://bmjopen.bmj.com/site/about/guidelines.xhtml

Study participants and site staff, except pharmacy staff, were blinded to treatment allocation. The clinician delivering the treatment injection was not blinded but was not involved in further trial-specific assessment of the participant. The trial statistician and data entry personnel were not blinded to the treatment allocation. The remaining members of the trial management team, including the staff conducting the qualitative interviews, were blinded to treatment allocation until after data analysis was complete.

Adalimumab/Placebo Injection

As per their treatment allocation, participants received either: intra-articular injection of adalimumab (160mg in 3.2mL for the first injection, 80mg in 1.6ml for second injection); or placebo (normal saline [0.9% sodium chloride] 3.2mL for the first injection and 1.6mL for the second injection). Full details of the injection delivery have been reported previously[20] and are described here in brief.

Appointments were coordinated so that participants received their first injection within approximately 2 weeks of randomisation, and second injection 2-3 weeks after the first (unless the participant declined, tested positive for tuberculosis or hepatitis B surface antigen, or had a related grade 3 or above adverse event). The injection was given into the anterior shoulder joint space in the rotator cuff interval where there is maximal inflammation of the capsule and synovium [21], under guided ultrasound by an appropriately qualified practitioner.

The adalimumab/placebo injection was dispensed by the local site pharmacy and sealed in identical sized and sealed opaque packaging. Preparation of the adalimumab/placebo injection took place in a clinic room/area separate from the participant to ensure the participant remained blinded to treatment allocation. Both adalimumab and placebo have a similar appearance; therefore, the two treatments are indistinguishable. The skin at the site of injection of adalimumab/placebo was infiltrated with local anaesthetic to reduce the pain of the injection as per local practice.

All participants received a written physiotherapy advice leaflet providing education and advice about frozen shoulder and pain management [4]. The advice leaflet also included simple self-guided exercises, which participants could use to increase shoulder joint mobilisation, once the early pain-predominant stage reduced. Participants were advised they may seek other forms of treatment during the follow-up period of the trial but were informed that they should use usual routes (e.g., through GP referral) to do so. Participants were also advised that they could seek corticosteroid injection but were asked to wait until after the 3-month follow-up period.

For peer review only - http://bmjopen.bmj.com/site/about/guidelines.xhtml

For peer review only

Outcome measures

Feasibility success criteria

To determine the feasibility of a definitive randomised controlled trial, the pre-specified success criteria were:

- Feasibility to recruit: ≥33% of potentially eligible patients with frozen shoulder screened eligible for recruitment.
- Success of consent process: ≥33% of eligible participants consented.
- Intervention delivery: ≥75% of participants receive 1st injection as randomised within specified timeframe.

Outcomes

In addition, we collected outcome measures at baseline and at 3-months to assess the feasibility of collecting these in a future definitive trial and to obtain the variability estimates required for estimation of the sample size of a definitive trial. Patient-reported outcomes, collected via online/paper questionnaire, included shoulder pain and function measured using the SPADI scale (primary outcome for definitive trial) [22, 23]; sub-domains of pain (SPADI 5-item pain subscale), function (SPADI 8-item disability subscale) [22, 23]; shoulder range of motion (Participant Shoulder Movement Questionnaire); psychological factors (Fear-Avoidance Belief Questionnaire) [24]; pain self-efficacy questionnaire [25]; sleep disturbance (Insomnia Severity Index) [26]; patient global impression of change [27]; return to desired activities; additional health resource use for index shoulder. At baseline and the 3 month face-to-face follow up appointment, a blinded assessor measured (using manual goniometer) range of shoulder movement.

Adverse events

The safety profile of adalimumab is well known, with the most common adverse reactions being mild injection site reactions. The Common Terminology Criteria for Adverse Events (CTCAE) v5.0 was used to guide recording of any adverse events including grading of the event. Only clinician-assessed adverse events, graded 3 and above, occurring during the trial for each participant, from their consent until the 3 month follow up, related to the trial medication (adalimumab/placebo) were recorded.

Sample size

The main feasibility objective and therefore the basis of the sample size estimate was participant recruitment per centre. The target sample size was 84 participants, equivalent of 1 to 2 participants

For peer review only - http://bmjopen.bmj.com/site/about/guidelines.xhtml

per month per site over 12 months. Seventy is the recommended minimum target sample size when including an estimate of the SD in an external pilot trial [28]. The sample size was increased to 84 to increase precision of the estimate of the standard deviation of SPADI at 3 months, the proposed primary outcome for the definitive trial, and to take into account possible attrition (based on an attrition rate of 15%).

Statistical analysis

Feasibility outcomes were reported as numbers and percentages. Baseline characteristics and outcome measures were reported using descriptive statistics, separately per group and overall, using either the mean and SD for continuous variables and number and percentage of participants in each group for categorical variables.

Embedded qualitative study

We planned to interview a purposive sample of up to 15 participants (or until we reach data saturation) to provide variability for age, gender, ethnicity and geographical representation. A qualitative researcher (CS), blinded to treatment allocation, conducted telephone interviews using a semi-structured interview guide with open-ended questions. Interviews were audio-taped and transcribed verbatim. Data were analysed following Braun and Clarke's thematic analysis method [29]. Interviews were coded using NVivo qualitative software Version 12 and categorised into themes. Participant narrative quotes were used to illustrate the themes.

Patient and public involvement

Patient representatives were involved in the design, conduct and reporting of study, including reviewing of patient-facing study materials (e.g. patient information sheet, physiotherapy advice booklet and patient follow-up questionnaires), and through involvement of the trial management and trial oversight committees.

RESULTS

Regulatory approvals were obtained on 22nd December 2021. Patient screening and enrolment began 31st May 2022 and ended 7th February 2023. Delays in obtaining site approvals due to the continuing impact of COVID-19 on staffing levels meant that site opening and thus patient screening was delayed, resulting in the recruitment period being just over eight months as opposed to the planned 12. Delays in local approvals at sites also meant we were only able to open four of the planned five sites as stipulated in the trial protocol.

For peer review only - http://bmjopen.bmj.com/site/about/guidelines.xhtml

Feasibility objectives

Feasibility to recruit

Of the 156 patients screened, 39 (25%) were eligible for the trial. The main reasons screened patients were not eligible was because they had other shoulder disorders (e.g., inflammatory arthritis, rotator cuff disorders, glenohumeral joint instability) (n=45/117; 38.5%); were no longer in the pain-predominant stage of frozen shoulder (n=39/117; 33.3%); had frozen shoulder due to significant shoulder trauma or other causes (e.g. recent breast cancer surgery) (n=11/117; 9.4%); had bilateral early stage frozen shoulder (n=7/117; 6%); or had received corticosteroid injection for shoulder pain within the last 12 weeks to either shoulder (n=5/117; 4.3%) (Figure 1).

Success of consent process:

Of the 39 patients eligible for the trial, 29 (74%) declined to participate, the main reasons being they already had a treatment preference for receiving steroid injection (n=13/29; 44.8%), did not wish to be randomised (n=7/29; 24%), or already had a treatment preference for not receiving an injection (n=4/29; 13.8%). In addition, one patient who was eligible was not consented due to close of trial recruitment. This resulted in nine (n=9/39; 23%) eligible patients being consented into the trial (Figure 1); four were randomised to receive adalimumab injection and five to placebo injection. Baseline characteristics are summarised in Table 1. Participants had a mean age of 54.9 (SD 5.6) years and three (33.3%) of the nine participants were women; all were white British. The average (mean) duration of symptoms was 15.4 weeks (SD 9.0). The mean SPADI score at baseline (primary outcome for definitive trial) was 70.8 (SD 16.6) for the adalimumab group and 55.4 (SD 21.5) for the placebo group.

Intervention delivery

All participants received their treatment as allocated. The mean time from randomisation to first injection was 12.3 days for the adalimumab group and 7.2 days for the placebo group. One participant in the adalimumab group received their injection 18 days post randomisation as they were not available until then. The mean time from receiving 1st to 2nd injection was similar for adalimumab (18.5 days) and placebo (20.8 days) (Table 2).

Patient retention and outcome measure data collection

Data for patient-reported outcomes and clinician-assessed range of shoulder motion are shown in Tables S1-3 (supplementary material). The completion rates at baseline and 3-month were 100% (n=9/9) for both patient reported outcomes, completed by questionnaire, and clinician assessed range

For peer review only - http://bmjopen.bmj.com/site/about/guidelines.xhtml

of shoulder motion. Between group difference were not calculated as only nine participants were recruited into the trial. Figure S1 (supplementary material) shows the change from baseline and at 3 months for shoulder pain and function (measured using the SPADI scale) per participant for adalimumab and placebo injection.

Adverse events

No graded 3 or above, adverse events occurred during the trial related to the trial medication (adalimumab/placebo).

Embedded qualitative study

Eight participants took part in the telephone interviews (four from each injection group), that included six males and two females with an average age of 55.5 years (range 46 to 62 years). Characteristics of those who consented and were interviewed are summarised in Table S4 (supplementary material). The average duration of interviews was 24 minutes (range 14 to 31 minutes). Five participants had received both injections at the time of interviews. Four key themes emerged: 1) experiences with pain-predominant stage of frozen shoulder; 2) perceptions about participating in the AFF trial; 3) perceptions about the trial processes; and 4) perceptions about the effects of injections. Themes and illustrative quotes are presented in Table 3.

Experiences with pain-predominant phase of frozen shoulder

This theme describes the impact of the pain-predominant phase of the frozen shoulder. Most participants reported severe levels of pain, particularly with certain movements or during sleep. The shoulder pain was described as 'very acute', 'annoying', 'disruptive', 'excruciating', or 'completely unbearable'. Participants also discussed how their everyday function was limited (e.g. difficulties with getting dressed or driving). Sleep was substantially affected by pain, with participants reporting shorter sleep times and poorer sleep quality. They frequently woke up in the night due to severe pain while changing position.

Perceptions about participating in the trial

This theme describes participants' views on participating in the AFF trial. Overall, participants were keen to find a solution to their shoulder problem. Severe pain, reduced function and sleep, and previous treatments such as steroid injections or physiotherapy that were not helpful were other reasons that influenced their decision to take part. Participants also reported altruistic reasons to benefit others with similar problems. Their expectations on taking part were to get adalimumab

For peer review only - http://bmjopen.bmj.com/site/about/guidelines.xhtml

injection, pain relief and improved function. Participants also expressed a clear understanding and acceptance of the randomisation process, mainly attributing to the trial-related information provided to them. Having a 50% chance of getting the placebo injection was a concern. However, participants discussed various reasons, including long waiting time for steroid injections, positive expectations about adalimumab, and flexibility to access standard care (if AFF trial injections didn't work) that helped them take a 'balanced view' to participate.

Perceptions about trial processes

This theme describes participant views about trial processes, including provision of information, delivery of injections, outcome assessments, and their overall experience. Participants described that all trial-related information such as randomisation and delivery of injections was communicated effectively, with opportunities to clarify their queries and make a decision. Injection appointments were scheduled quickly, and participants were well-informed and supported during the sessions. Many reported discomfort at the time of injections. Participants perceived the outcome measurements (clinician-assessed measurements and self-reports) as relevant to their condition and had no issues completing them. They also highlighted their positive experiences with the trial personnel at study sites and reported an overall positive experience of participating in the AFF trial.

Perceived effects of adalimumab and placebo injections

Two participants who received adalimumab injections reported improvements in pain and/or shoulder movements. Another participant reported some pain relief after the first injection. Pain remained unchanged and movement decreased after the second injection. One participant who had received first injection only, reported improvement in movement. Two participants who received placebo injections reported increased pain after their first injection that persisted after the second injection. Of the two participants who had only their first injection, one reported noticeable improvement in pain and movement; the other reported increased pain. Two participants (one from each group) were concerned about the increased pain after the injections. Other concerns were expectations about the long-term effects of adalimumab and continued access to physiotherapy within the trial.

DISCUSSION

Our findings show that a definitive trial to assess whether an intra-articular injection of adalimumab (anti-TNF) can reduce pain and improve function in people with early-stage frozen shoulder was not feasible within the NHS musculoskeletal and related physiotherapy services that participated in the AFF trial. Only a quarter of the people screened were eligible to take part in this feasibility study; of those eligible, only nine consented to be randomised. Key themes from the semi-structured interviews

For peer review only - http://bmjopen.bmj.com/site/about/guidelines.xhtml

provided a useful insight into the trial and focused on the participant experiences during the pain-dominant stage of frozen shoulder and their perceptions about trial participation, trial processes and outcomes following injection.

One of the main reasons people were ineligible to take part in the AFF trial was because they were no longer in the pain-predominant phase of frozen shoulder by the time they were seen by the physiotherapist. People no longer being in the pain-predominant phase of frozen shoulder is indicative of long waiting lists and time taken to be seen across NHS musculoskeletal and related physiotherapy services and was a major barrier in terms of trial feasibility. Long waiting times is a problem across the NHS, particularly during and post the COVID-19 pandemic [30]; the period in which the AFF trial was recruiting and not specific to people with frozen shoulder. The four NHS sites that participated in the AFF trial represented several different musculoskeletal and physiotherapy service delivery approaches, with approaches also changing during and post the COVID-19 pandemic. Several of these sites also included a small number of First Contact Practitioners (FCPs) embedded with primary care. However, given the very small number of trial participants and small number of FCPs, we are not able to determine if moving intervention delivery earlier in the patient pathway would have improved recruitment. In addition, our early feasibility work to identify potential trial sites identified lack of local site pharmacy (i.e. for storage and dispensing of adalimumab) and delivery of injection under ultrasound guidance was a major barrier to conducting the AFF trial in primary care and should be considered for any future trial.

We employed a number of different recruitment strategies to try and maximise recruitment at sites. This included sending targeted trial information packs to GPs and health care professionals, such as FCPs, within a 15 mile radius of each recruiting site. We promoted the trial at scientific conferences targeting clinicians in the shoulder community and created a short trial promotional video for the Chartered Society of Physiotherapists. We also promoted the trial on social media with input from our patient representative, and through patient organisations such as the Dupuytren's Society and Diabetes UK to target groups in whom the incidence of frozen shoulder is higher [1, 15].

Another main barrier to feasibility of the trial was because some people, who were eligible and approached to take part in the AFF trial, already had a treatment preference for receiving a steroid injection and declined to participate. We did consider the use of corticosteroid injection as opposed to placebo when designing the trial; however, given the available evidence showing that steroid injection only leads to short-term benefit [8, 31], we decided to use a placebo injection of saline (0.9%

For peer review only - http://bmjopen.bmj.com/site/about/guidelines.xhtml

sodium chloride) as the comparator. Unlike steroid (triamcinolone), which is a white suspension, saline has the same appearance to adalimumab meant we were able to blind participants as to their treatment allocation. This was especially important given the subjective nature of some of the outcomes we planned to assess in the definitive trial, should this study have shown to be feasible. Interestingly, one of the main findings from our interviews was that participants understood the concept of randomisation and accepted the possibility of receiving placebo.

Despite not meeting the feasible objectives in terms of recruitment and success of the consent process, it was feasible to deliver the intervention in line with the trial protocol and all participants completed patient-reported and clinician assessed outcomes at 3 months. Data from the semi-structured interviews showed that participants found the overall experience of the trial was positive. They participated in the AFF trial considering it as an opportunity to find a solution to their shoulder problem and also to benefit others.

Strengths

To our knowledge, AFF is the first study to assess the feasibility of conducting a large multi-centre trial to test the effectiveness of a new treatment for people with early stage, pain-predominant frozen shoulder. This is important given the evidence for the limited effectiveness for treatments currently offered to people with frozen shoulder [7, 8, 10, 11, 32]. The drug adalimumab has a very strong safety profile, having been used in over 5 million people, more than 25,000 trial participants and approved for nine different disorders [33]. We used a targeted injection approach using ultrasound guidance, which was standardised across sites and meant we were able to maintain blinding of trial participants and members of trial team at sites. Despite the small number of participants recruited into the trial the semi-structured interviews provided a useful insight into patient experiences with the early painful phase of frozen shoulder and their perceptions about being involved in the AFF trial. AFF is one of the very few feasibility trials of upper limb conditions that include a qualitative component.

Limitations

A limitation of this study is that all participants were white British with an average age of 55 years. Therefore, the population recruited were not truly representative of the general population or those people affected by frozen shoulder [34], despite recruiting from sites within inner cities with a more diverse population demographic. In addition, while all nine participants recruited into the trial received both their first and second injection within the specific timeframe, it is unclear whether this would be same for larger definitive trial with more pressure on treatment appointment slots. Similarly,

For peer review only - http://bmjopen.bmj.com/site/about/guidelines.xhtml

while all participants completed both patient-reported and clinician-assessed outcomes at 3 months, it is unlikely this would be same for larger trial with longer follow-up. In a recent trial of corticosteroid injection and physiotherapy in people with a rotator cuff disorder, carried out across 20 NHS musculoskeletal services, the attrition rate at 6 and 12 months was 13% [35]. It is also uncertain whether data saturation was reached from the eight semi-structured interviews and, therefore. these findings may not be generalisable and should be interpreted within the context of the AFF trial.

Conclusion

The evidence from this feasibility study demonstrated that a definitive trial was not feasible within the NHS musculoskeletal and related physiotherapy services that participated in the AFF trial. This was largely due to difficulties in identifying patients while still in early-stage frozen shoulder and patient expectations regarding steroid injection. However, it was feasible to deliver the injection in line with trial protocol and to collect both patient and clinician assessed outcome data. Our interview findings indicated positive experiences from participants who took part in the trial.

Acknowledgments: This study is coordinated by the UKCRC registered Oxford Clinical Trials Research Unit (OCTRU) at the University of Oxford. It followed their Standard Operating Procedures, ensuring compliance with the principles of Good Clinical Practice and the Declaration of Helsinki and any appliable regulatory requirements. Special thanks go Nicola Kenealy as trial manager and Sue Dutton as co-applicant (now retired) and members of the Trial Oversight Committee: Ben Fisher (chair; University of Birmingham), Jim Wiggle (Patient Representative), Rebecca Evans (University of Bristol), Mike Thomas (Heatherwood and Wexham Park NHS Hospitals Trust), Anju Jaggi (Royal National Orthopaedic Hospital NHS Trust).

Collaborators: The Anti-Freaze-F study collaborating principal investigators were Vincent Gallagher (East Sussex Healthcare NHS Trust), Neil Smith (Sandwell and West Birmingham Hospital NHS Trust), Alun Yewlett (United Lincolnshire Hospitals NHS Trust), Paul Barker (University Hospitals Birmingham).

Contributors: SH and JN are co-chief investigators and co-lead applicants. AR, MF, JP and SL are co-applicants on the grant awarded by the National Institute of Health Research - Research for Patient Benefit Programme (Project reference: 201031) and were involved in design and conduct of the study, and interpretation of data. CS led the qualitative study, FE and SM conducted the statistical analysis

For peer review only - http://bmjopen.bmj.com/site/about/guidelines.xhtml

and AE and EAF provided trial management expertise. SH, JN and CS led on writing the manuscript. All authors read, commented and approved the final version.

Funding: This report is independent research funded by the National Institute of Health Research - Research for Patient Benefit Programme (Project reference: 201031) The views expressed in this publication are those of the author(s) and not necessarily those of the NIHR or the Department of Health and Social Care. 180 Life Sciences provided funding for the purchase of adalimumab, the investigational medicinal product used in this trial.

Disclaimer: The views expressed are those of the author(s) and not necessarily those of the NHS, the NIHR or the Department of Health.

Competing interests: Professor Sir Marc Feldman and Professor Jagdeep Nanchahal are co-founders and hold equity in 180 Life Sciences, the company funding the purchase of adalimumab, the investigational medicinal product to be used in this trial.

Ethics approval: Health Research Authority North East - Newcastle & North Tyneside 1 (REC 21/NE/0214).

Data sharing statement: Summary results data will be available on the trial registration databases within 12 months of the end of the trial. Requests for data (anonymised trial participant level data) will be provided at the end of the trial to external researchers who provide a methodologically sound proposal to the trial team (and who will be required to sign a data sharing access agreement with the Sponsor) and in accordance with the NIHR guidance. Participant consent for this was included in the informed consent form for the study.

For peer review only

REFERENCES

1. Dawson, J., Shepperd, S., Carr, A., *An overview of factors relevant to undertaking research and reviews on the effectiveness of treatment for frozen shoulder.* Shoulder and Elbow,, 2010. **2**: p. 232-237.
2. Walker-Bone, K., et al., *Prevalence and impact of musculoskeletal disorders of the upper limb in the general population.* Arthritis Rheum, 2004. **51**(4): p. 642-51.
3. Hand, C., et al., *Long-term outcome of frozen shoulder.* J Shoulder Elbow Surg, 2008. **17**(2): p. 231-6.
4. Rangan, A., N. Hanchard, and C. McDaid, *What is the most effective treatment for frozen shoulder?* Bmj, 2016. **354**: p. i4162.
5. Hanchard, N.C., et al., *Evidence-based clinical guidelines for the diagnosis, assessment and physiotherapy management of contracted (frozen) shoulder: quick reference summary.* Physiotherapy, 2012. **98**(2): p. 117-20.
6. Ryan, V., et al., *The pathophysiology associated with primary (idiopathic) frozen shoulder: A systematic review.* BMC Musculoskelet Disord, 2016. **17**(1): p. 340.
7. Buchbinder, R., et al., *Oral steroids for adhesive capsulitis.* Cochrane Database Syst Rev, 2006(4): p. Cd006189.
8. Buchbinder, R., S. Green, and J.M. Youd, *Corticosteroid injections for shoulder pain.* Cochrane Database Syst Rev, 2003(1): p. Cd004016.
9. Rangan, A., et al., *Management of adults with primary frozen shoulder in secondary care (UK FROST): a multicentre, pragmatic, three-arm, superiority randomised clinical trial.* Lancet, 2020. **396**(10256): p. 977-989.
10. Page, M.J., et al., *Electrotherapy modalities for adhesive capsulitis (frozen shoulder).* Cochrane Database Syst Rev, 2014(10): p. Cd011324.
11. Page, M.J., et al., *Manual therapy and exercise for adhesive capsulitis (frozen shoulder).* Cochrane Database Syst Rev, 2014(8): p. Cd011275.
12. Bunker, T.D., et al., *Expression of growth factors, cytokines and matrix metalloproteinases in frozen shoulder.* J Bone Joint Surg Br, 2000. **82**(5): p. 768-73.
13. Lho, Y.M., et al., *Inflammatory cytokines are overexpressed in the subacromial bursa of frozen shoulder.* J Shoulder Elbow Surg, 2013. **22**(5): p. 666-72.
14. Bunker, T.D. and P.P. Anthony, *The pathology of frozen shoulder. A Dupuytren-like disease.* J Bone Joint Surg Br, 1995. **77**(5): p. 677-83.
15. Smith, S.P., V.S. Devaraj, and T.D. Bunker, *The association between frozen shoulder and Dupuytren's disease.* J Shoulder Elbow Surg, 2001. **10**(2): p. 149-51.
16. Hand, G.C., et al., *The pathology of frozen shoulder.* J Bone Joint Surg Br, 2007. **89**(7): p. 928-32.
17. Nanchahal, J., et al., *Anti-Tumour Necrosis Factor Therapy for Dupuytren's Disease: A Randomised Dose Response Proof of Concept Phase 2a Clinical Trial.* EBioMedicine, 2018. **33**: p. 282-288.
18. Nanchahal, J., et al., *Anti-tumour necrosis factor therapy for early-stage Dupuytren's disease (RIDD): a phase 2b, randomised, double-blind, placebo-controlled trial.* Lancet Rheumatol, 2022. **4**(6): p. E407-e416.
19. British Elbow and Shoulder Society, B.O.A., Royal College of Surgeons, *Commissioning guide: subacromial shoulder pain*. 2014.
20. Hopewell, S., et al., *Anti-TNF (adalimumab) injection for the treatment of adults with frozen shoulder during the pain predominant stage protocol for a multi-centre, randomised, double blind, parallel group, feasibility trial [version 2; peer review: 2 approved]. .* NIHR Open Res, 2023. **2:28**.
21. Sun, Y., et al., *The Effect of Corticosteroid Injection Into Rotator Interval for Early Frozen Shoulder: A Randomized Controlled Trial.* Am J Sports Med, 2018. **46**(3): p. 663-670.

For peer review only - http://bmjopen.bmj.com/site/about/guidelines.xhtml

22. Roach, K.E., et al., *Development of a shoulder pain and disability index.* Arthritis Care Res, 1991. **4**(4): p. 143-9.
23. Williams, J.W., Jr., D.R. Holleman, Jr., and D.L. Simel, *Measuring shoulder function with the Shoulder Pain and Disability Index.* J Rheumatol, 1995. **22**(4): p. 727-32.
24. Mintken, P.E., et al., *Psychometric properties of the Fear-Avoidance Beliefs Questionnaire and Tampa Scale of Kinesiophobia in patients with shoulder pain.* Arch Phys Med Rehabil, 2010. **91**(7): p. 1128-36.
25. Nicholas, M.K., B.E. McGuire, and A. Asghari, *A 2-item short form of the Pain Self-efficacy Questionnaire: development and psychometric evaluation of PSEQ-2.* J Pain, 2015. **16**(2): p. 153-63.
26. Bastien, C.H., A. Vallieres, and C.M. Morin, *Validation of the Insomnia Severity Index as an outcome measure for insomnia research.* Sleep Med, 2001. **2**(4): p. 297-307.
27. Kamper, S.J., C.G. Maher, and G. Mackay, *Global rating of change scales: a review of strengths and weaknesses and considerations for design.* J Man Manip Ther, 2009. **17**(3): p. 163-70.
28. Teare, M.D., et al., *Sample size requirements to estimate key design parameters from external pilot randomised controlled trials: a simulation study.* Trials, 2014. **15**: p. 264.
29. Braun, V.C., V., *Using thematic analysis in psychology.* Qualitative Research in Psychology, 2006. **3**(2): p. 77-101.
30. Limb, M., *Long NHS waits and rising living costs are harming people's health, show ONS data.* Bmj, 2023. **380**: p. 479.
31. Wang, W., et al., *Effectiveness of corticosteroid injections in adhesive capsulitis of shoulder: A meta-analysis.* Medicine (Baltimore), 2017. **96**(28): p. e7529.
32. Maund, E., et al., *Management of frozen shoulder: a systematic review and cost-effectiveness analysis.* Health Technol Assess, 2012. **16**(11): p. 1-264.
33. US Food and Drug Adminstration: www.accessdata.fda.gov/drugsatfda_docs/label/2018/125057s406lbl.pdf [accessed 05 July 2023].
34. *Office of National Statistics. Ethnicity https://www.ons.gov.uk/peoplepopulationandcommunity/culturalidentity/ethnicity [accessed 05 July 2023].*
35. Hopewell, S., et al., *Progressive exercise compared with best practice advice, with or without corticosteroid injection, for the treatment of patients with rotator cuff disorders (GRASP): a multicentre, pragmatic, 2 × 2 factorial, randomised controlled trial.* Lancet, 2021. **398**(10298): p. 416-428.

For peer review only

For peer review only - http://bmjopen.bmj.com/site/about/guidelines.xhtml

Figure 1: Participant flow diagram

[insert figure 1]

For peer review only

Table 1: Baseline characteristics by intervention group

	Adalimumab (n=4)	Placebo (n=5)	Total (n=9)
**Age**[1]	57.5 (5.9)	52.8(5.0)	54.9 (5.6)
**Sex**[2]			
Male	3 (75.0%)	3 (60.0%)	6 (66.7%)
Female	1 (25.0%)	2 (40.0%)	3 (33.3%)
**Ethnicity**[2]			
White British	4 (100.0%)	5 (100.0%)	9 (100.0%)
**Handedness**[2]			
Left	2 (50.0%)	1 (20.0%)	3 (33.3%)
Right	2 (50.0%)	4 (80.0%)	6 (66.7%)
**Duration of symptoms** (weeks)[1]	19.5 (13.1)	12.2 (2.3)	15.4 (9.0)
**Affected shoulder**[2]			
Left	3 (75.0%)	2 (40.0%)	5 (55.6%)
Right	1 (25.0%)	3 (60.0%)	4 (44.4%)
**BMI (kg/m^2)**[1]	29.5 (5.8)	30.3 (5.3)	30.0 (5.2)
**Smoking status**[2]			
Never smoked	4 (100.0%)	3 (60.0%)	7 (77.8%)
Former smoker	0	2 (40.0%)	2 (22.2%)
If ever smoked, number of cigarettes/day[1]	0	12 (11.3)	12 (11.3)
**Dupuytren's disease**[2]			
No	4 (100.0%)	4 (80.0%)	8 (88.9%)
Yes	0	1 (20.0%)	1 (11.1%)
**Diabetes**[2]			
Type 1	0	1 (20.0%)	1 (11.1%)
Type 2	1 (25.0%)	2 (40.0%)	3 (33.3%)
No	3 (75.0%)	2 (40.0%)	5 (55.6%)

[1] Summaries are mean (SD)

[2] Summaries are n (%)

Table 2: Injection delivery

	Adalimumab (n=4)	Placebo (n=5)	Overall (n=9)
**First injection given**[1]	4 (100%)	5 (100%)	9 (100%)
Time from randomisation to first injection (days)[2]	12.3 (4.8)	7.2 (4.1)	9.4 (4.9)
Injection administered?[1]			
Yes – fully	4 (100%)	5 (100%)	9 (100%)
**Second injection given**[1]	4 (100%)	5 (100%)	9 (100%)
Time from first to second injection (days)[2]	18.5 (3.3)	20.8 (5.9)	19.8 (4.8)
Injection adminstered?[1]			
Yes – fully	4 (100%)	5 (100%)	9 (100%)

[1] Summaries are n (%)

[2] Summaries are mean (SD)

For peer review only - http://bmjopen.bmj.com/site/about/guidelines.xhtml

Table 3: Themes and participant quotes from qualitative interviews

Themes	Narrative quotes
Experiences with pain-predominant phase of frozen shoulder	• *'I always put childbirth as a 10, so I always come back from that really, so I would say it was kind of like an eight to nine*[49-year-old female, Adalimumab]<br>• *'It affects when I pretty much … every day, whatever I'm doing'* [53-year-old male, Placebo]<br>• *' it just doesn't help me sleeping. I'm getting about three hours and max four hours at nights…'* [58-year-old male, Placebo]
Perceptions about participation in the trial	• *'…if it's gonna help me and help other people then, yeah, it's something I'd do'* [46-year-old male, Placebo]<br>• *'My first thought was yes, I was very happy to, primarily because the current treatment hadn't been particularly successful and I was in still quite a bit of pain and struggling to sleep very well and I thought the fact that another option was being made available, I was more than comfortable to participate'* [58-year-old male, Adalimumab]<br>• *'my expectation really was that if I did get the drug it was likely that I would see some kind of improvement'* [49-year-old female, Adalimumab]<br>• *'I hoped that I got the drug and not the placebo and it helped'* [61-year-old male, Adalimumab]<br>• *I accepted that that's now how drug trials work and, therefore, I knew there was a possibility that I may not be receiving the drug'* [58-year-old male, Adalimumab]<br>• *'I mean initially I was concerned because if I was the placebo I felt that potentially I was gonna be, well maybe three months further down the line having had the placebo and no benefit. And then I'd have to go back to square one so I was slightly anxious about that. But I was reassured that it, you know, it wasn't stopping me then getting further treatment if I hadn't had any response to the injections. And I guess, this is a bit sceptical of me that waiting lists are what they are but I probably would have been waiting three months for a steroid injection anyway. So, I didn't think it as a disadvantage to do the trial really. If it worked, great, and if it didn't, then I'd-- like I say, I'd go back to the GP'* [57-year-old female, Placebo]
Perceptions about trial processes	• *'I couldn't fault the information I was given to be honest, they were very good'* [46-year-old male, Placebo]<br>• *'I just went in had the injection. It wasn't very pleasant. It was quite…quite painful. It did make me feel a little bit queasy at the time'* [62-year-old male, Adalimumab]<br>• *'I was happy to do that because I understand that they have to gauge any improvement in my shoulder movements over time. So, yeah, I was happy to complete those movements'* [53-year-old male, Placebo]<br>• *'I mean it all went well. I was seen on time, everything was ready. Everything went to plan'* [61-year-old male, Adalimumab]<br>• *'I'm really delighted that I'm part of the trial now because I'm seeing the benefit of it already. So, I'm very … I'm thrilled. I feel very lucky… I mean I do feel-- I feel honoured and I feel like they've treated me so well'* [57-year-old female, Placebo]
Perceptions about effects of adalimumab and placebo injections	After first and second adalimumab injections<br>• *'I think there was a noticeable improvement from the first injection. Perhaps a slightly… bit of an extra improvement after the second one. But I think it was quite noticeable following that first injection'* [58-year-old male, Adalimumab]<br>• *Perhaps a slightly… bit of an extra improvement after the second one. But I think it was quite noticeable following that first injection'.. I definitely have got significant reduction in the pain. I've got more what I would say is a tightness in the muscular areas now, so this almost feels as though where I was on the old regime – pre-trial'* [58-year-old male, Adalimumab]<br><br>After first and second placebo injections<br>• *'once I had the first injection, my arm … the actual … the pain actually increased quite significantly in my shoulder for a good 7 to 10 days. And I was having to take*

For peer review only

For peer review only - http://bmjopen.bmj.com/site/about/guidelines.xhtml

	strong painkillers to sort of manage that. It's actually settled down a bit now' [53-year-old female, Placebo]<br>• *'…there wasn't any significant increase in pain after the second injection…I've not noticed any real improvement since I had the second injection…I still do get the discomfort that I've got in my shoulder'* [53-year-old female, Placebo]

For peer review only

For peer review only - http://bmjopen.bmj.com/site/about/guidelines.xhtml

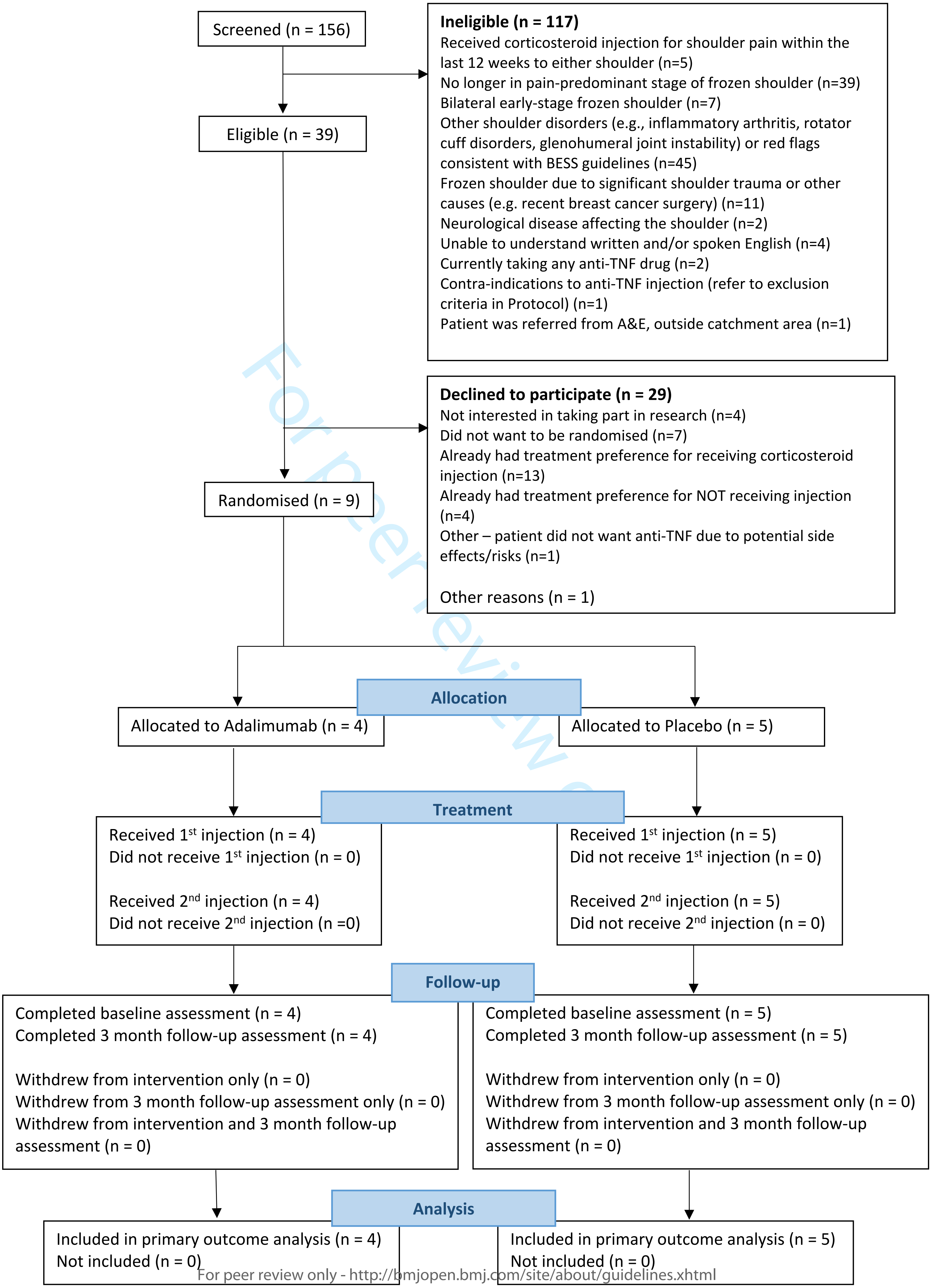
Screened (n = 156)
Ineligible (n = 117)
Received corticosteroid injection for shoulder pain within the last 12 weeks to either shoulder (n=5)
No longer in pain-predominant stage of frozen shoulder (n=39)
Bilateral early-stage frozen shoulder (n=7)
Other shoulder disorders (e.g., inflammatory arthritis, rotator cuff disorders, glenohumeral joint instability) or red flags consistent with BESS guidelines (n=45)
Frozen shoulder due to significant shoulder trauma or other causes (e.g. recent breast cancer surgery) (n=11)
Neurological disease affecting the shoulder (n=2)
Unable to understand written and/or spoken English (n=4)
Currently taking any anti-TNF drug (n=2)
Contra-indications to anti-TNF injection (refer to exclusion criteria in Protocol) (n=1)
Patient was referred from A&E, outside catchment area (n=1)
Eligible (n = 39)
Declined to participate (n = 29)
Not interested in taking part in research (n=4)
Did not want to be randomised (n=7)
Already had treatment preference for receiving corticosteroid injection (n=13)
Already had treatment preference for NOT receiving injection (n=4)
Other – patient did not want anti-TNF due to potential side effects/risks (n=1)
Other reasons (n = 1)
Randomised (n = 9)
Allocation
Allocated to Adalimumab (n = 4)
Allocated to Placebo (n = 5)
Treatment
Received 1st injection (n = 4)
Did not receive 1st injection (n = 0)
Received 2nd injection (n = 4)
Did not receive 2nd injection (n =0)
Received 1st injection (n = 5)
Did not receive 1st injection (n = 0)
Received 2nd injection (n = 5)
Did not receive 2nd injection (n = 0)
Follow-up
Completed baseline assessment (n = 4)
Completed 3 month follow-up assessment (n = 4)
Withdrew from intervention only (n = 0)
Withdrew from 3 month follow-up assessment only (n = 0)
Withdrew from intervention and 3 month follow-up assessment (n = 0)
Completed baseline assessment (n = 5)
Completed 3 month follow-up assessment (n = 5)
Withdrew from intervention only (n = 0)
Withdrew from 3 month follow-up assessment only (n = 0)
Withdrew from intervention and 3 month follow-up assessment (n = 0)
Analysis
Included in primary outcome analysis (n = 4)
Not included (n = 0)
Included in primary outcome analysis (n = 5)
Not included (n = 0)

Supplementary materials (online only)

Figure S1: SPADI score: change from baseline and at 3 months

a) **Total SPADI Scores at baseline and 3 months for each participant**

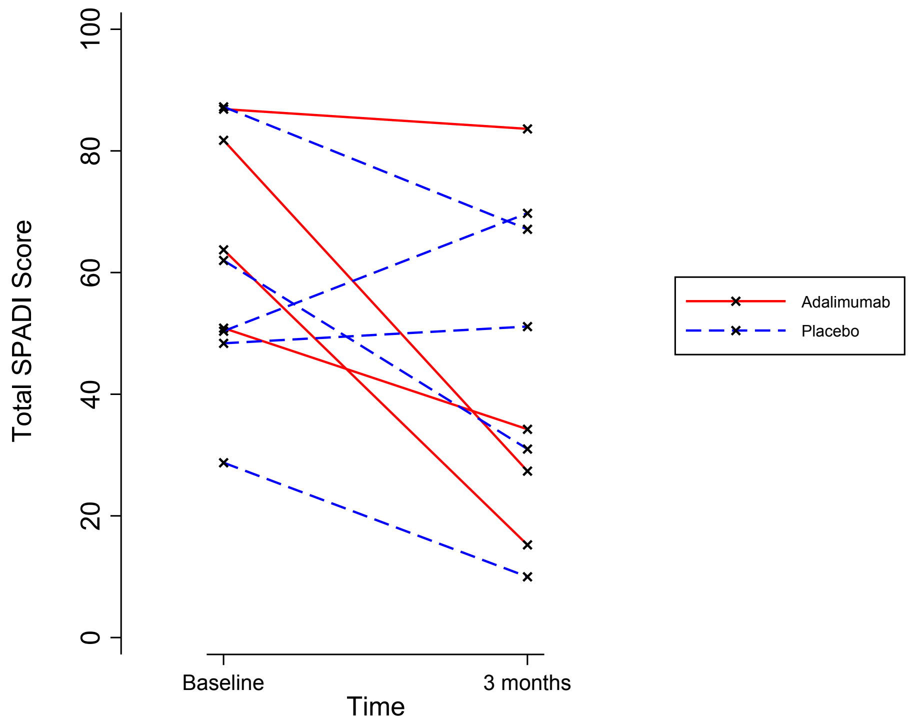

b) **SPADI Pain Subscale Scores at baseline and 3 months for each participant**

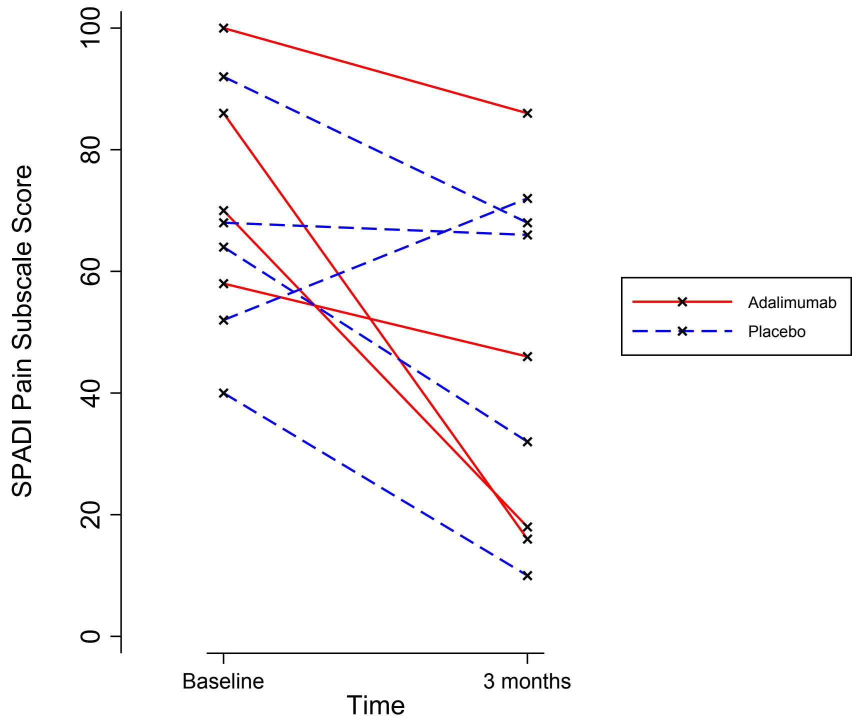

c) SPADI Function Subscale Scores at baseline and 3 months for each participant

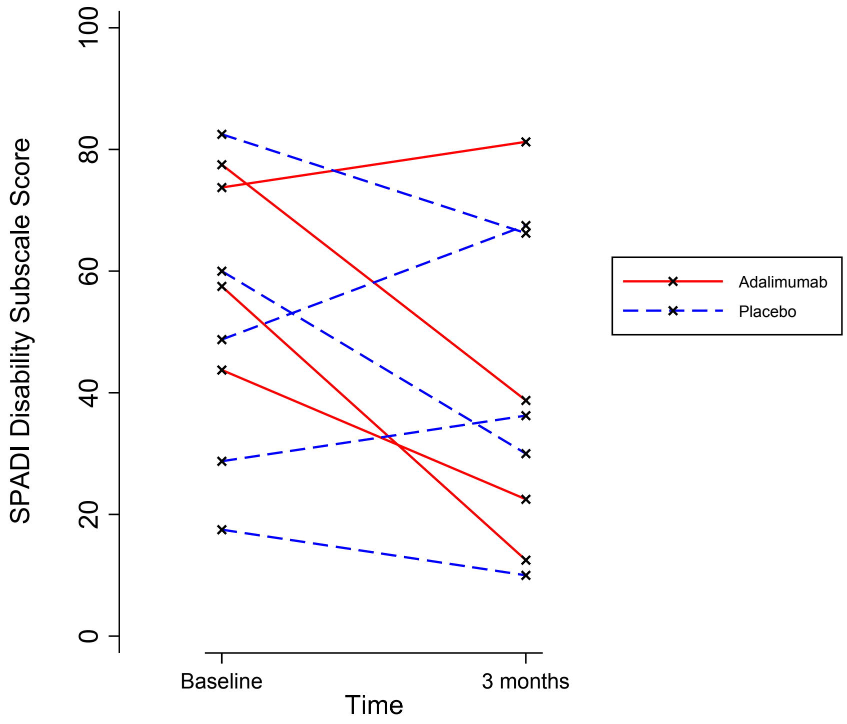

review only

Table S1: Secondary patient-reported outcomes

		Adalimumab[1] (n=4)	Placebo[1] (n=5)
SPADI Total score	Baseline	70.8 (16.6)	55.4 (21.5)
	3 months	40.1 (30.0)	45.8 (25.3)
SPADI pain (subscale)	Baseline	78.5 (18.4)	63.2 (19.5)
	3 months	41.5 (32.7)	49.6 (27.3)
SPADI disability (subscale)	Baseline	63.1 (15.6)	47.5 (25.7)
	3 months	38.8 (30.3)	42 (24.7)
FAB-Q	Baseline	17.3 (3.3)	17.4 (3.6)
	3 months	9.3 (6.2)	12.8 (5.9)
PSEQ – short-form	Baseline	9 (3.6)	9.2 (1.9)
	3 months	10.5 (1.3)	8.2 (2.8)
ISI	Baseline	17.8 (5.)	17.6 (4.0)
	3 months	9.8 (6.7)	13.2 (7.6)
Return to desired activities	Baseline	9 (4.5)	9.4 (2.7)
	3 months	5.5 (3.8)	8.4 (3.6)
Global impression of change	3 months	2.3 (2.2)	0 (3.0)

[1] *Summaries are mean (SD)*

SPADI: Shoulder Pain And Disability Index; FAB-Q: Fear-Avoidance Belief Questionnaire; PSEQ: Pain Self-Efficacy Questionnaire; ISI: Insomnia Severity Index

For peer review only

For peer review only - http://bmjopen.bmj.com/site/about/guidelines.xhtml

Table S2: Clinician-assessed range of shoulder motion

	Adalimumab[1] (n=4)	Placebo[1] (n=5)
**Flexion**		
Baseline	71.3 (26.3)	101.6 (24.7)
3 months	112.5 (54.8)	140 (25.7)
**Extension**		
Baseline	26.3 (18.0)	27.6 (9.5)
3 months	30.0 (8.2)	40.6 (9.8)
**Abduction**		
Baseline	50.0 (30.3)	69.0 (26.6)
3 months	105.0 (56.1)	115.0 (42.7)
**Internal rotation**		
Baseline	23.8 (18.9)	40.2 (40.8)
3 months	21.3 (17.0)	41 (37.8)
**External rotation**		
Baseline	22.5 (8.7)	15.0 (16.9)
3 months	28.8 (6.3)	55.0 (16.2)

[1] *Summaries are mean (SD) degrees of shoulder motion*

Table S3: Patient reported range of shoulder motion

	Adalimumab[1] (n=4)	Placebo[1] (n=5)
**Flexion**		
Baseline	102.3 (21.3)	112.4 (21.3)
3 months	117.3 (38.4)	145.6 (22.9)
**Extension**		
Baseline	26.3 (6.9)	23.7 (8.5)
3 months	23.5 (7.0)	36.5 (7.0)
**Abduction**		
Baseline	81.3 (35.4)	99.3 (27.0)
3 months	79.3 (45.7)	134.2 (52.5)
**Internal rotation**		
Baseline	18.5 (19.8)	29 (29.9)
3 months	51.0 (15.6)	72.3 (19.4)
**External rotation**		
Baseline	35.3 (15.7)	24.8 (14.5)
3 months	35.5 (9.8)	37.0 (24.0)

[1] *Summaries are mean (SD) degrees of shoulder motion*

For peer review only

For peer review only - http://bmjopen.bmj.com/site/about/guidelines.xhtml

Table S4 Characteristics of interview participants

Trial group	Gender	Ethnicity
Placebo	Male	White British
Placebo [1st injection]	Female	White British
Placebo	Male	White British
Placebo [1st injection]	Male	White British
Adalimumab	Male	White British
Adalimumab	Female	White British
Adalimumab	Male	White British
Adalimumab [1st injection]	Male	White British

For peer review only

For peer review only - http://bmjopen.bmj.com/site/about/guidelines.xhtml

Table 2 | CONSORT checklist of information to include when reporting a pilot trial

Section/topic and item No	Standard checklist item	Extension for pilot trials	Page No where item is reported
**Title and abstract**			
1a	Identification as a randomised trial in the title	Identification as a pilot or feasibility randomised trial in the title	1
1b	Structured summary of trial design, methods, results, and conclusions (for specific guidance see CONSORT for abstracts)	Structured summary of pilot trial design, methods, results, and conclusions (for specific guidance see CONSORT abstract extension for pilot trials)	2
**Introduction**			
Background and objectives:			
2a	Scientific background and explanation of rationale	Scientific background and explanation of rationale for future definitive trial, and reasons for randomised pilot trial	4-5
2b	Specific objectives or hypotheses	Specific objectives or research questions for pilot trial	4-5
**Methods**			
Trial design:			
3a	Description of trial design (such as parallel, factorial) including allocation ratio	Description of pilot trial design (such as parallel, factorial) including allocation ratio	5
3b	Important changes to methods after trial commencement (such as eligibility criteria), with reasons	Important changes to methods after pilot trial commencement (such as eligibility criteria), with reasons	N/A
Participants:			
4a	Eligibility criteria for participants		6
4b	Settings and locations where the data were collected		6
4c		How participants were identified and consented	6
Interventions:			
5	The interventions for each group with sufficient details to allow replication, including how and when they were actually administered		7
Outcomes:			
6a	Completely defined prespecified primary and secondary outcome measures, including how and when they were assessed	Completely defined prespecified assessments or measurements to address each pilot trial objective specified in 2b, including how and when they were assessed	8
6b	Any changes to trial outcomes after the trial commenced, with reasons	Any changes to pilot trial assessments or measurements after the pilot trial commenced, with reasons	N/A
6c		If applicable, prespecified criteria used to judge whether, or how, to proceed with future definitive trial	8
Sample size:			
7a	How sample size was determined	Rationale for numbers in the pilot trial	8-9
7b	When applicable, explanation of any interim analyses and stopping guidelines		
Randomisation:			
Sequence generation:			
8a	Method used to generate the random allocation sequence		6
8b	Type of randomisation; details of any restriction (such as blocking and block size)	Type of randomisation(s); details of any restriction (such as blocking and block size)	6
Allocation concealment mechanism:			
9	Mechanism used to implement the random allocation sequence (such as sequentially numbered containers), describing any steps taken to conceal the sequence until interventions were assigned		6
Implementation:			
10	Who generated the random allocation sequence, enrolled participants, and assigned participants to interventions		6
Blinding:			
11a	If done, who was blinded after assignment to interventions (eg, participants, care providers, those assessing outcomes) and how		6-7
11b	If relevant, description of the similarity of interventions		6-7
Analytical methods:			
12a	Statistical methods used to compare groups for primary and secondary outcomes	Methods used to address each pilot trial objective whether qualitative or quantitative	9
12b	Methods for additional analyses, such as subgroup analyses and adjusted analyses	Not applicable	N/A

BMJ: first published as 10.1136/bmj.i5239 on 24 October 2016. Downloaded from http://www.bmj.com/ on 26 July 2023 at Bodleian Libraries of the University of Oxford. Protected by copyright.

Table 2 | CONSORT checklist of information to include when reporting a pilot trial

Section/topic and item No	Standard checklist item	Extension for pilot trials	Page No where item is reported
**Results**			
Participant flow (a diagram is strongly recommended):			
13a	For each group, the numbers of participants who were randomly assigned, received intended treatment, and were analysed for the primary outcome	For each group, the numbers of participants who were approached and/or assessed for eligibility, randomly assigned, received intended treatment, and were assessed for each objective	figure 1
13b	For each group, losses and exclusions after randomisation, together with reasons		figure 1
Recruitment:			
14a	Dates defining the periods of recruitment and follow-up		9
14b	Why the trial ended or was stopped	Why the pilot trial ended or was stopped	9
Baseline data:			
15	A table showing baseline demographic and clinical characteristics for each group		Table 1, p20
Numbers analysed:			
16	For each group, number of participants (denominator) included in each analysis and whether the analysis was by original assigned groups	For each objective, number of participants (denominator) included in each analysis. If relevant, these numbers should be by randomised group	Table 2, p20 page 10
Outcomes and estimation:			
17a	For each primary and secondary outcome, results for each group, and the estimated effect size and its precision (such as 95% confidence interval)	For each objective, results including expressions of uncertainty (such as 95% confidence interval) for any estimates. If relevant, these results should be by randomised group	Table 2, p20 page 10
17b	For binary outcomes, presentation of both absolute and relative effect sizes is recommended	Not applicable	N/A.
Ancillary analyses:			
18	Results of any other analyses performed, including subgroup analyses and adjusted analyses, distinguishing prespecified from exploratory	Results of any other analyses performed that could be used to inform the future definitive trial	N/A
Harms:			
19	All important harms or unintended effects in each group (for specific guidance see CONSORT for harms)		
19a		If relevant, other important unintended consequences	N/A.
**Discussion**			
Limitations:			
20	Trial limitations, addressing sources of potential bias, imprecision, and, if relevant, multiplicity of analyses	Pilot trial limitations, addressing sources of potential bias and remaining uncertainty about feasibility	14
Generalisability:			
21	Generalisability (external validity, applicability) of the trial findings	Generalisability (applicability) of pilot trial methods and findings to future definitive trial and other studies	13-14
Interpretation:			
22	Interpretation consistent with results, balancing benefits and harms, and considering other relevant evidence	Interpretation consistent with pilot trial objectives and findings, balancing potential benefits and harms, and considering other relevant evidence	13-14
22a		Implications for progression from pilot to future definitive trial, including any proposed amendments	13-14
**Other information**			
Registration:			
23	Registration number and name of trial registry	Registration number for pilot trial and name of trial registry	2
Protocol:			
24	Where the full trial protocol can be accessed, if available	Where the pilot trial protocol can be accessed, if available	6
Funding:			
25	Sources of funding and other support (such as supply of drugs), role of funders		16
26		Ethical approval or approval by research review committee, confirmed with reference number	16

BMJ: first published as 10.1136/bmj.i5239 on 24 October 2016. Downloaded from http://www.bmj.com/ on 26 July 2023 at Bodleian Libraries of the University of Oxford. Protected by copyright.

BMJ Open

Anti-TNF (adalimumab) injection for the treatment of pain predominant early-stage frozen shoulder: the Anti-Freaze-Feasibility randomised controlled trial.

Journal:	*BMJ Open*
Manuscript ID	bmjopen-2023-078273.R1
Article Type:	Original research
Date Submitted by the Author:	15-Feb-2024
Complete List of Authors:	Hopewell, Sally; University of Oxford, Oxford Clinical Trials Research Unit, Nuffield Department of Orthopaedics, Rheumatology and Musculoskeletal Sciences<br>Srikesavan, Cynthia; University of Oxford, Centre for Rehabilitation Research in Oxford, Nuffield Department of Orthopaedics, Rheumatology and Musculoskeletal Sciences; University of Exeter, College of Medicine and Health<br>Evans, Alison; University of Oxford, Kennedy Institute of Rheumatology, Nuffield Department of Orthopaedics, Rheumatology and Musculoskeletal Sciences<br>Er, Fema; University of Oxford, Oxford Clinical Trials Research Unit, Nuffield Department of Orthopaedics, Rheumatology and Musculoskeletal Sciences<br>Rangan, Amar; The James Cook University Hospital, South Tees Hospitals NHS Foundation Trust; University of York, Department of Health Sciences<br>Preece, Jane; Patient and Public Involvement Representative<br>Francis, Anne; University of Oxford, Oxford Clinical Trials Research Unit, Nuffield Department of Orthopaedics, Rheumatology and Musculoskeletal Science<br>Massa, M Sofia; University of Oxford, Oxford Clinical Trials Research Unit, Nuffield Department of Orthopaedics, Rheumatology and Musculoskeletal Sciences<br>Feldmann, Marc; University of Oxford, Kennedy Institute of Rheumatology, Nuffield Department of Orthopaedics, Rheumatology and Musculoskeletal Sciences<br>Lamb, Sarah; University of Exeter, College of Medicine and Health<br>Nanchahal, Jagdeep; The University of Oxford, Kennedy Institute of Rheumatology, Nuffield Department of Orthopaedics, Rheumatology and Musculoskeletal Sciences
**Primary Subject Heading**:	Rehabilitation medicine
Secondary Subject Heading:	Rheumatology
Keywords:	Patients, Shoulder < ORTHOPAEDIC & TRAUMA SURGERY, Musculoskeletal disorders < ORTHOPAEDIC & TRAUMA SURGERY

For peer review only - http://bmjopen.bmj.com/site/about/guidelines.xhtml

I, the Submitting Author has the right to grant and does grant on behalf of all authors of the Work (as defined in the below author licence), an exclusive licence and/or a non-exclusive licence for contributions from authors who are: i) UK Crown employees; ii) where BMJ has agreed a CC-BY licence shall apply, and/or iii) in accordance with the terms applicable for US Federal Government officers or employees acting as part of their official duties; on a worldwide, perpetual, irrevocable, royalty-free basis to BMJ Publishing Group Ltd ("BMJ") its licensees and where the relevant Journal is co-owned by BMJ to the co-owners of the Journal, to publish the Work in this journal and any other BMJ products and to exploit all rights, as set out in our licence.

The Submitting Author accepts and understands that any supply made under these terms is made by BMJ to the Submitting Author unless you are acting as an employee on behalf of your employer or a postgraduate student of an affiliated institution which is paying any applicable article publishing charge ("APC") for Open Access articles. Where the Submitting Author wishes to make the Work available on an Open Access basis (and intends to pay the relevant APC), the terms of reuse of such Open Access shall be governed by a Creative Commons licence – details of these licences and which Creative Commons licence will apply to this Work are set out in our licence referred to above.

Other than as permitted in any relevant BMJ Author's Self Archiving Policies, I confirm this Work has not been accepted for publication elsewhere, is not being considered for publication elsewhere and does not duplicate material already published. I confirm all authors consent to publication of this Work and authorise the granting of this licence.

For peer review only

Anti-TNF (adalimumab) injection for the treatment of pain predominant early-stage frozen shoulder: the Anti-Freeze-Feasibility randomised controlled trial.

Sally Hopewell[1], Cynthia Srikesavan[2,3], Alison Evans[4], Fema Er[1], Amar Rangan[5,6], Jane Preece[7], Anne Francis[1], Sofia Massa[1], Marc Feldmann[4], Sarah E Lamb[3], Jagdeep Nanchahal[4]

1. Oxford Clinical Trials Research Unit, Nuffield Department of Orthopaedics, Rheumatology and Musculoskeletal Sciences, University of Oxford, UK.
2. Centre for Rehabilitation Research in Oxford, Nuffield Department of Orthopaedics, Rheumatology and Musculoskeletal Sciences, University of Oxford, UK
3. University of Exeter Medical School, UK.
4. Kennedy Institute of Rheumatology, Nuffield Department of Orthopaedics, Rheumatology and Musculoskeletal Sciences, University of Oxford, UK.
5. James Cook University Hospital, South Tees Hospitals NHS Foundation Trust, Middlesbrough, UK.
6. Health Sciences and HYMS, University of York, UK
7. Patient and Public Involvement Representative, Kidderminster, UK

Correspondence to: Professor Sally Hopewell, Oxford Clinical Trials Research Unit / Centre for Statistics in Medicine, Nuffield Department of Orthopaedics, Rheumatology and Musculoskeletal Sciences, University of Oxford. Email: sally.hopewell@csm.ox.ac.uk

For peer review only

ABSTRACT

Objective: The Anti-Freaze-F (AFF) trial assessed the feasibility of conducting a definitive trial to determine whether intra-articular injection of adalimumab can reduce pain and improve function in people with pain-predominant early-stage frozen shoulder.

Design: Multi-centre, randomised feasibility trial, with embedded qualitative study.

Setting: Four UK NHS musculoskeletal and related physiotherapy services.

Participants: Adults ≥18 years with new episode of shoulder pain attributable to early-stage frozen shoulder.

Interventions: Participants were randomised (centralised computer generated 1:1 allocation) to either ultrasound guided intra-articular injection of: 1) adalimumab (160mg) or 2) placebo (saline [0.9% sodium chloride]). Participants and outcome assessors were blinded to treatment allocation. Second injection of allocated treatment (adalimumab 80mg) or equivalent placebo was administered 2-3 weeks later.

Primary feasibility objectives: 1) ability to screen and identify participants; 2) willingness of eligible participants to consent and be randomised; 3) practicalities of delivering the intervention; 4) standard deviation of the Shoulder Pain and Disability Index (SPADI) score and attrition rate at 3 months.

Results: Between 31 May 2022 and 7 February 2023, 156 patients were screened of whom 39 (25%) were eligible. The main reasons for ineligibility were other shoulder disorder (38.5%; n=45/117) or no longer in pain-predominant frozen shoulder (33.3%; n=39/117). Of the 39 eligible patients, nine (23.1%) consented to be randomised (adalimumab n=4; placebo n=5). The main reason patients declined was because they preferred receiving steroid injection (n=13). All participants received treatment as allocated. The mean time from randomisation to first injection was 12.3 (adalimumab) and 7.2 days (placebo). Completion rates for patient-reported and clinician-assessed outcomes were 100%.

Conclusion: This study demonstrated that current NHS musculoskeletal physiotherapy settings yielded only small numbers of participants, too few to make a trial viable. This was because many patients had passed the early stage of frozen shoulder or had already formulated a preference for treatment.

Trial registration: ISRCTN 27075727, EudraCT 2021-03509-23, ClinicalTrials.gov NCT05299242. (REC 21/NE/0214).

For peer review only - http://bmjopen.bmj.com/site/about/guidelines.xhtml

STRENGHTENS AND LIMITATIONS OF THIS STUDY

- The Anti-Freaze-F study was a pilot randomised controlled trial with an embedded qualitative study to test the effectiveness of a new treatment for people with early stage, pain-predominant frozen shoulder.
- Participants were randomised (centralised computer generated 1:1 allocation) to either ultrasound guided intra-articular injection of adalimumab or placebo injection. Participants and outcome assessors were blinded to treatment allocation.
- We assessed the feasibility of conducting a large multicentre randomised trial in terms of the ability to screen and identify participants, their willingness to consent and be randomised, the practicalities of delivering the intervention and attrition rates at 3 months.
- While all nine participants recruited into the trial received both their first and second injection within the specific timeframe, it is unclear whether this would be same for larger definitive trial.
- Similarly, while all participants completed both patient-reported and clinician-assessed outcomes at 3 months, it is unlikely this would be same for larger trial with longer follow-up.

For peer review only - http://bmjopen.bmj.com/site/about/guidelines.xhtml

INTRODUCTION

Frozen shoulder (adhesive capsulitis) is a common and extremely painful and debilitating condition. Affected individuals struggle with activities of daily living and significant sleep disturbances as a result of severe pain [1]. It affects about 9% of people in the United Kingdom (UK) aged 25-64 years [2], and 20% develop the same problem in the other shoulder [3]. Frozen shoulder may develop as a primary condition or secondarily following surgery or trauma.

The classic description of the development of frozen shoulder is of three overlapping phases [4, 5]. The initial pain-predominant inflammatory phase is characterised by constant pain and difficulty sleeping and lasts between 3 and 9 months. This progresses to a stiffness-predominant fibrotic phase, with progressive restriction of motion, particularly external rotation and elevation of the shoulder, and impairment of function, and lasts between 4 and 12 months. The pain changes from being constant to being manifest at the end of range of motion and of reduced intensity. Over the final phase, there is a gradual improvement in range of motion and stiffness over a 12 to 48-month period, although pain may persist with end-range movements. The average duration of the condition is 30 months (range 1 to 3.5 years) although full resolution of symptoms does not always occur [6].

The aetiology of frozen shoulder is poorly understood and consequently there is no consensus on the optimal treatment. The majority of patients with early-stage pain predominant frozen shoulder are managed in primary care or at primary care interface musculoskeletal services by physiotherapists and general practitioners. During this stage, standard treatment consists of rest, advice, analgesics, physiotherapy, and corticosteroid injections to address the symptoms. Current treatments available to patients with frozen shoulder are of limited efficacy. Two Cochrane reviews concluded that whilst oral steroid or local steroid injections lead to short-term benefit in pain and range of motion, the effects are not maintained beyond 6 weeks [7, 8]. A more recent systematic review (based on data from 5 randomised trials) also found some benefit of corticosteroid injection compared to placebo but this this pain relief was not sustained in the long term [9]. Other Cochrane reviews concluded that there is no evidence that physiotherapy or ultrasound therapy are beneficial [10], and that manual therapy with exercise is less effective than corticosteroid injection in the short-term [11]. The UK FROST compared the effects of physiotherapy plus corticosteroid injection, manipulation under anaesthesia with a steroid injection, and arthroscopic capsular release supplemented with manipulation [12]. None of the treatments were found to be clinically superior.

Our study (Anti-Freaze-F) was designed to specifically target people with early-stage pain predominant

For peer review only

frozen shoulder. We assessed the feasibility of conducting a large multicentre randomised trial to test whether giving an intra-articular injection of adalimumab (a drug targeting the inflammatory mediator tumour necrosis factor [TNF]), can reduce pain and prevent the condition from getting worse, if given during the early pain predominant stage.

Approximately 50% of people with frozen shoulder also develop Dupuytren's disease [13]. Dupuytren's disease has a prominent genetic component and in a study of twins the heritability was estimated at 80% [14]. We have recently confirmed that there is a significant genetic correlation between Dupuytren's disease and frozen shoulder [15]. The pathology of the two conditions has several similarities [16, 17]. A systematic review of the pathophysiology of frozen shoulder identified the presence of fibrosis and the role of inflammation [6]. The affected tissues are infiltrated by immune cells and there are elevated levels of pro-inflammatory cytokines, including tumour necrosis factor (TNF) [18, 19], with myofibroblasts driving the development of fibrosis [16]. In a dose-ranging proof of concept phase 2a followed by a 2b clinical trial (RIDD trial) we found that injection of anti-TNF (adalimumab) directly into Dupuytren's nodules led to significant downregulation of the myofibroblast phenotype and reduction in nodule hardness (primary endpoint) and nodule size on ultrasound scan (secondary endpoint) [20, 21].

Objectives

The aim of the Anti-Freaze-F (AFF) trial was to assess the feasibility of conducting a large randomised controlled trial to assess whether an intra-articular injection of adalimumab (anti-TNF) can reduce pain and improve function in people with early-stage frozen shoulder. The primary objectives were to assess the:

- ability to screen and identify potential participants with pain predominant early stage frozen shoulder;
- willingness of eligible participants to consent and be randomised to intervention;
- practicalities of delivering the intervention, including time to first injection and number of participants receiving second injection;
- standard deviation (SD) of the Shoulder Pain and Disability Index (SPADI) score and attrition rate at 3 months (i.e. 12 weeks) post-randomisation in order to estimate the sample size for a definitive trial.

Secondary objectives were to assess the follow up rates and viability of patient-reported outcome measures and clinician-assessed shoulder range of motion at 3 months (i.e. 12 weeks).

For peer review only - http://bmjopen.bmj.com/site/about/guidelines.xhtml

The objectives of the embedded qualitative study were to explore the participant experience of being recruited to the AFF trial, the treatment received and follow-up schedule, and to understand what helps participant recruitment to the trial intervention.

METHODS

Study design

A multi-centre, randomised, double-blind, parallel group, feasibility trial with an embedded qualitative study.

Setting

Participants were recruited from four NHS musculoskeletal services and their related physiotherapy services, with treatment delivered within these services or local secondary care dependant on the local service provision.

Study participants

Participants were eligible if they were aged 18 years and above with a new episode of shoulder pain attributable to pain-predominant stage of frozen shoulder (i.e. within approximately three to nine months of onset of symptoms), which was diagnosed clinically using the criteria set out in the British Elbow and Shoulder Society (BESS) guidelines [22]. In line with the trial protocol, imaging, including plain radiographs, may be used to help confirm a diagnosis of frozen shoulder by excluding other pathology such as glenohumeral arthritis as per standard NHS care. However, is not always necessary and hence routine radiology was not as part of the trial procedures [23]. Patients were excluded if they had frozen shoulder secondary to significant shoulder trauma; other shoulder disorders (e.g., inflammatory arthritis, rotator cuff disorders, glenohumeral joint instability) or with red flags consistent with the criteria set out in the BESS guidelines [22]; bilateral early stage frozen shoulder; had received corticosteroid injection for shoulder pain in the last 12 weeks; were currently taking any anti-TNF drug, or being treated with coumarin anticoagulants, such as warfarin; had significant renal or hepatic impairment, or had contra-indications to anti-TNF injection. Detailed inclusion and exclusion criteria are described in the trial protocol [23]. Patients who met the eligibility criteria and wanted to participate were approached for written informed consent by a research facilitator trained in Good Clinical Practice at each participating site. Ethics approval was obtained from the National Research Ethics Committee and MHRA (REC 21/NE/0214). All participants underwent serological testing to check for latent tuberculosis (TB) and hepatitis B surface antigen. The blood tests were performed during the baseline assessment or at the time the participant attended for their first

For peer review only - http://bmjopen.bmj.com/site/about/guidelines.xhtml

injection appointment (depending on the local site provision). The risks of reactivation following a single injection are low. If any participants had shown a positive serology test result, they would have been referred to their local infectious diseases service and would not have received the second injection.

Randomisation

Consented participants were randomly assigned to receive either: 1) intra-articular injection of adalimumab; or 2) placebo injection (saline [0.9% sodium chloride], both under ultrasound guidance. Randomisation (1:1) was done by the research facilitator at each site using the centralised randomisation service provided by the Oxford Clinical Trials Research Unit once the patient was enrolled and baseline assessment and questionnaire completed. Randomisation was computer-generated and stratified by study site using a variable block size (variable block sizes of 2, 4 and 6 in a ratio 1:2:1) to ensure the participants from each study site had equal chance of receiving either intervention.

Blinding

Study participants and site staff, except pharmacy staff, were blinded to treatment allocation. The clinician delivering the treatment injection was not blinded but was not involved in further trial-specific assessment of the participant. The trial statistician and data entry personnel were not blinded to the treatment allocation. The remaining members of the trial management team, including the staff conducting the qualitative interviews, were blinded to treatment allocation until after data analysis was complete.

Adalimumab/Placebo Injection

As per their treatment allocation, participants received either: intra-articular injection of adalimumab (160mg in 3.2mL for the first injection, 80mg in 1.6ml for second injection); or placebo (normal saline [0.9% sodium chloride] 3.2mL for the first injection and 1.6mL for the second injection). Full details of the injection delivery have been reported previously [23] and are described here in brief.

Appointments were coordinated so that participants received their first injection within approximately 2 weeks of randomisation, and second injection 2-3 weeks after the first (unless the participant declined, tested positive for tuberculosis or hepatitis B surface antigen, or had a related grade 3 or above adverse event). The injection was given into the anterior shoulder joint space in the rotator cuff interval where there is maximal inflammation of the capsule and synovium [24], under guided ultrasound by an appropriately qualified practitioner.

For peer review only - http://bmjopen.bmj.com/site/about/guidelines.xhtml

The adalimumab/placebo injection was dispensed by the local site pharmacy and sealed in identical sized and sealed opaque packaging. Preparation of the adalimumab/placebo injection took place in a clinic room/area separate from the participant to ensure the participant remained blinded to treatment allocation. Both adalimumab and placebo have a similar appearance; therefore, the two treatments are indistinguishable. The skin at the site of injection of adalimumab/placebo was infiltrated with local anaesthetic to reduce the pain of the injection as per local practice. Injection details, including success of the intra-articular injection (i.e. fully or partial), was recorded on a trial specific injection treatment log.

All participants received a written physiotherapy advice leaflet providing education and advice about frozen shoulder and pain management [4]. The advice leaflet also included simple self-guided exercises, which participants could use to increase shoulder joint movements, once the early pain-predominant stage reduced. Participants were advised they may seek other forms of treatment during the follow-up period of the trial but were informed that they should use usual routes (e.g., through GP referral) to do so. Participants were also advised that they could seek corticosteroid injection but were asked to wait until after the 3-month follow-up period.

Outcome measures

Feasibility success criteria

To determine the feasibility of a definitive randomised controlled trial, the pre-specified success criteria were:

- Feasibility to recruit: ≥33% of potentially eligible patients with frozen shoulder screened eligible for recruitment.
- Success of consent process: ≥33% of eligible participants consented.
- Intervention delivery: ≥75% of participants receive 1st injection as randomised within specified timeframe.

In addition, we collected outcomes at baseline and at 3-months to assess the feasibility of collecting these in a future definitive trial and to obtain the variability estimates required for estimation of the sample size of a definitive trial. Patient-reported outcomes, collected via online/paper questionnaire, included shoulder pain and function measured using the SPADI scale (primary outcome for definitive trial) [25, 26]; sub-domains of pain (SPADI 5-item pain subscale), function (SPADI 8-item disability subscale) [25, 26]; shoulder range of motion (Participant Shoulder Movement Questionnaire);

For peer review only - http://bmjopen.bmj.com/site/about/guidelines.xhtml

psychological factors (Fear-Avoidance Belief Questionnaire) [27]; pain self-efficacy questionnaire [28]; sleep disturbance (Insomnia Severity Index) [29]; patient global impression of change [30]; return to desired activities; and additional health resource use. At baseline and the 3 month face-to-face follow-up appointment, a blinded assessor measured the shoulder movements including active flexion, extension, abduction internal and external rotations, using a manual goniometer.

Adverse events

The safety profile of adalimumab is well known, with the most common adverse reactions being mild injection site reactions. The Common Terminology Criteria for Adverse Events (CTCAE) v5.0 was used to guide recording of any adverse events including grading of the event. Only clinician-assessed adverse events, graded 3 and above, occurring during the trial for each participant, from their consent until the 3 month follow up, related to the trial medication (adalimumab/placebo) were recorded.

Sample size

The main feasibility objective and therefore the basis of the sample size estimate was participant recruitment per centre. The target sample size was 84 participants, equivalent of 1 to 2 participants per month per site over 12 months. Seventy is the recommended minimum target sample size when including an estimate of the SD in an external pilot trial [31]. The sample size was increased to 84 to increase precision of the estimate of the standard deviation of SPADI at 3 months, the proposed primary outcome for the definitive trial, and to consider possible attrition based on an attrition rate of 15%.

Statistical analysis

Feasibility outcomes were reported as numbers and percentages. Baseline characteristics and outcome measures were reported using descriptive statistics, separately per group and overall, using either the mean and SD for continuous variables and number and percentage of participants in each group for categorical variables.

Embedded qualitative study

We planned to interview a purposive sample of up to 15 participants (or until we reach data saturation) to provide variability for age, gender, ethnicity, and geographical representation. A qualitative researcher (CS), blinded to treatment allocation, conducted the telephone interviews using a semi-structured interview guide with open-ended questions. Interviews were audio-taped and transcribed verbatim. Data were analysed following Braun and Clarke's thematic analysis method [32].

For peer review only - http://bmjopen.bmj.com/site/about/guidelines.xhtml
For peer review only

Interviews were coded using NVivo qualitative software Version 12 and categorised into themes. Participant narrative quotes were used to illustrate the themes.

Patient and public involvement

Patient representatives were involved in the design, conduct and reporting of study, including reviewing of patient-facing study materials (e.g. patient information sheet, physiotherapy advice booklet and patient follow-up questionnaires), and through involvement of the trial management and trial oversight committees.

RESULTS

Regulatory approvals were obtained on 22nd December 2021. Patient screening and enrolment began 31st May 2022 and ended 7th February 2023; completion of the 3-month outcome assessment and follow-up ended 9th May 2023. Delays in obtaining site approvals due to the continuing impact of COVID-19 on staffing levels meant that site opening and thus patient screening was delayed, resulting in the recruitment period being just over eight months as opposed to the planned 12 months. Delays in local approvals at sites also meant we were only able to open four of the planned five sites as stipulated in the trial protocol.

Feasibility objectives

Feasibility to recruit

Of the 156 patients screened, 39 (25%) were eligible for the trial. The main reasons screened patients were not eligible was because they had other shoulder disorders (e.g., inflammatory arthritis, rotator cuff disorders, glenohumeral joint instability) (n=45/117; 38.5%); were no longer in the pain-predominant stage of frozen shoulder (n=39/117; 33.3%); had frozen shoulder due to significant shoulder trauma or other causes (e.g. recent breast cancer surgery) (n=11/117; 9.4%); had bilateral early stage frozen shoulder (n=7/117; 6.0%); or had received corticosteroid injection for shoulder pain within the last 12 weeks to either shoulder (n=5/117; 4.3%) (Figure 1).

Success of consent process:

Of the 39 patients eligible for the trial, 29 (74.4%) declined to participate, the main reasons being they already had a treatment preference for receiving steroid injection (n=13/29; 44.8%), did not wish to be randomised (n=7/29; 24.1%), or already had a treatment preference for not receiving an injection (n=4/29; 13.8%). In addition, one patient who was eligible was not consented due to close of trial recruitment. This resulted in nine (n=9/39; 23.1%) eligible patients being consented into the trial

For peer review only - http://bmjopen.bmj.com/site/about/guidelines.xhtml

(Figure 1); four were randomised to receive adalimumab injection and five to placebo injection. Baseline characteristics are summarised in Table 1. Participants had a mean age of 54.9 (SD 5.6) years and three (33.3%) of the nine participants were women; all were white British. The average (mean) duration of symptoms was 15.4 weeks (SD 9.0). The mean SPADI score at baseline (primary outcome for definitive trial) was 70.8 (SD 16.6) for the adalimumab group and 55.4 (SD 21.5) for the placebo group.

Intervention delivery

All participants received their treatment as allocated and in line with the trial protocol. The mean time from randomisation to first injection was 12.3 days for the adalimumab group and 7.2 days for the placebo group. One participant in the adalimumab group received their injection 18 days post randomisation as they were not available until then. The mean time from receiving 1st to 2nd injection was similar for adalimumab (18.5 days) and placebo (20.8 days) (Table 2).

Patient retention and outcome measure data collection

Data for patient-reported outcomes and clinician-assessed range of shoulder motions are shown in Tables S1-3 (supplementary material). The completion rates at baseline and 3-month were 100% (n=9/9) for both patient-reported outcomes, completed by questionnaire, and clinician-assessed shoulder motion. Between-group difference were not calculated as only nine participants were recruited into the trial. Figure S1 (supplementary material) shows the change from baseline and at 3 months for shoulder pain and function (measured using the SPADI scale) per participant for adalimumab and placebo injection.

Adverse events

No graded 3 or above, adverse events occurred during the trial related to the trial medication (adalimumab/placebo).

Embedded qualitative study

Eight participants took part in the telephone interviews (four from each injection group), that included six males and two females with an average age of 55.5 years (range 46 to 62 years). The average duration of interviews was 24 minutes (range 14 to 31 minutes). Five participants had received both injections at the time of interviews. Four key themes emerged: 1) experiences with pain-predominant stage of frozen shoulder; 2) perceptions about participating in the AFF trial; 3) perceptions about the

For peer review only - http://bmjopen.bmj.com/site/about/guidelines.xhtml

For peer review only

trial processes; and 4) perceptions about the effects of injections. Themes and illustrative quotes are presented in Table 3.

Experiences with pain-predominant phase of frozen shoulder

This theme describes the impact of the pain-predominant phase of the frozen shoulder. Most participants reported severe levels of pain, particularly with certain movements or during sleep. The shoulder pain was described as 'very acute', 'annoying', 'disruptive', 'excruciating', or 'completely unbearable'. Participants also discussed how their everyday function was limited (e.g. difficulties with getting dressed or driving). Sleep was substantially affected by pain, with participants reporting shorter sleep times and poorer sleep quality. They frequently woke up in the night due to severe pain while changing position.

Perceptions about participating in the trial

This theme describes participants' views on participating in the AFF trial. Overall, participants were keen to find a solution to their shoulder problem. Severe pain, reduced function and sleep, and previous treatments such as steroid injections or physiotherapy that were not helpful were other reasons that influenced their decision to take part. Participants also reported altruistic reasons to benefit others with similar problems. Their expectations on taking part were to get adalimumab injection, pain relief and improved function. Participants also expressed a clear understanding and acceptance of the randomisation process, mainly attributing to the trial-related information provided to them. Having a 50% chance of getting the placebo injection was a concern. However, participants discussed various reasons, including long waiting time for steroid injections, positive expectations about adalimumab, and flexibility to access standard care (if AFF trial injections didn't work) that helped them take a 'balanced view' in participating.

Perceptions about trial processes

This theme describes participant views about trial processes, including provision of information, delivery of injections, outcome assessments, and their overall experience. Participants described that all trial-related information such as randomisation and delivery of injections was communicated effectively, with opportunities to clarify their queries and make a decision. Injection appointments were scheduled quickly, and participants were well-informed and supported during the sessions. Many reported discomfort at the time of injections. Participants perceived the outcome measurements (clinician-assessed measurements and self-reports) as relevant to their condition and

For peer review only - http://bmjopen.bmj.com/site/about/guidelines.xhtml

had no issues completing them. They also highlighted their positive experiences with the trial personnel at study sites and reported an overall positive experience of participating in the AFF trial.

Perceived effects of adalimumab and placebo injections

Two participants who received adalimumab injections reported improvements in pain and/or shoulder movements. Another participant reported some pain relief after the first injection. Pain remained unchanged and movement decreased after the second injection. One participant who had received first injection only, reported improvement in movement. Two participants who received placebo injections reported increased pain after their first injection that persisted after the second injection. Of the two participants who had only their first injection, one reported noticeable improvement in pain and movement; the other reported increased pain. Two participants (one from each group) were concerned about the increased pain after the injections. Other concerns were expectations about the long-term effects of adalimumab and continued access to physiotherapy within the trial.

DISCUSSION

Our findings show that a definitive trial to assess whether an intra-articular injection of adalimumab (anti-TNF) can reduce pain and improve function in people with early-stage frozen shoulder was not feasible within the NHS musculoskeletal and related physiotherapy services that participated in the AFF trial. Only a quarter of the people screened were eligible to take part in this feasibility study; of those eligible, only nine consented to be randomised. Key themes from the semi-structured interviews provided a useful insight into the trial and focused on the participant experiences during the pain-dominant stage of frozen shoulder and their perceptions about trial participation, trial processes and outcomes following injection.

One of the main reasons people were ineligible to take part in the AFF trial was because they were no longer in the pain-predominant phase of frozen shoulder by the time they were seen by the physiotherapist. People no longer being in the pain-predominant phase of frozen shoulder is indicative of long waiting lists and time taken to be seen across NHS musculoskeletal and related physiotherapy services and was a major barrier in terms of trial feasibility. Long waiting times is a problem across the NHS, particularly during and after the COVID-19 pandemic [33]; the period in which the AFF trial was recruiting and not specific to people with frozen shoulder. The four NHS sites that participated in the AFF trial represented several different musculoskeletal and physiotherapy service delivery approaches, with approaches also changing during and post the COVID-19 pandemic. Several of these sites also included a small number of First Contact Practitioners (FCPs) embedded with primary care. However, given the very small number of trial participants and small number of FCPs, we are not able

For peer review only - http://bmjopen.bmj.com/site/about/guidelines.xhtml

to determine if moving intervention delivery earlier in the patient pathway would have improved recruitment. In addition, our early feasibility work to identify potential trial sites identified lack of local site pharmacy (i.e. for storage and dispensing of adalimumab) and delivery of injection under ultrasound guidance was a major barrier to conducting the AFF trial in primary care and should be considered for any future trial.

We employed a number of different recruitment strategies to try and maximise recruitment at sites. This included sending targeted trial information packs to GPs and health care professionals, such as FCPs, within a 15-mile radius of each recruiting site. We promoted the trial at scientific conferences targeting clinicians in the shoulder community and created a short trial promotional video for the Chartered Society of Physiotherapists. We also promoted the trial on social media with input from our patient representative, and through patient organisations such as the Dupuytren's Society and Diabetes UK to target groups in whom the incidence of frozen shoulder is higher [1, 13].

Another main barrier to feasibility of the trial was because some people, who were eligible and approached to take part in the AFF trial, already had a treatment preference for receiving a steroid injection and declined to participate. We did consider the use of corticosteroid injection as opposed to placebo when designing the trial; however, given the available evidence showing that steroid injection only leads to short-term benefit [8, 9], we decided to use a placebo injection of saline (0.9% sodium chloride) as the comparator. Unlike steroid (triamcinolone), which is a white suspension, saline has the same appearance to adalimumab meant we were able to blind participants as to their treatment allocation. This was especially important given the subjective nature of some of the outcomes we planned to assess in the definitive trial, should this study have shown to be feasible. Interestingly, one of the main findings from our interviews was that participants understood the concept of randomisation and accepted the possibility of receiving placebo.

Despite not meeting the feasible objectives in terms of recruitment and success of the consent process, it was feasible to deliver the intervention in line with the trial protocol and all participants completed patient-reported and clinician assessed outcomes at 3 months. Data from the semi-structured interviews showed that participants found the overall experience of the trial was positive. They participated in the AFF trial considering it as an opportunity to find a solution to their shoulder problem and also to benefit others.

Strengths

For peer review only - http://bmjopen.bmj.com/site/about/guidelines.xhtml

To our knowledge, AFF is the first study to assess the feasibility of conducting a large multi-centre trial to test the effectiveness of a new treatment for people with early stage, pain-predominant frozen shoulder. This is important given the evidence for the limited effectiveness for treatments currently offered to people with frozen shoulder [7, 8, 10, 11, 34]. The drug adalimumab has a very strong safety profile, having been used in over 5 million people, more than 25,000 trial participants and approved for nine different disorders [35]. We used a targeted injection approach using ultrasound guidance, which was standardised across sites and meant we were able to maintain blinding of trial participants and members of trial team at sites. Despite the small number of participants recruited into the trial, our interviews provided a useful insight into patient experiences with the early painful phase of frozen shoulder and their perceptions about being involved in the AFF trial. AFF is one of the very few feasibility trials of upper limb conditions that include a qualitative component.

Limitations

A limitation of this study is that all participants were white British with an average age of 55 years. Therefore, the population recruited were not truly representative of the general population or those people affected by frozen shoulder [36], despite recruiting from sites within inner cities with a more diverse population demographic. In addition, while all nine participants recruited into the trial received both their first and second injection within the specific timeframe, it is unclear whether this would be same for larger definitive trial with more pressure on treatment appointment slots. Similarly, while all participants completed both patient-reported and clinician-assessed outcomes at 3 months, it is unlikely this would be same for larger trial with longer follow-up. In a recent trial of corticosteroid injection and physiotherapy in people with a rotator cuff disorder, carried out across 20 NHS musculoskeletal services, the attrition rate at 6 and 12 months was 13% [37]. It is also uncertain whether data saturation was reached from the eight semi-structured interviews and, therefore. these findings may not be generalisable and should be interpreted only within the context of the AFF trial.

Conclusion

Our findings demonstrated that a definitive trial was not feasible within the NHS musculoskeletal and related physiotherapy services that participated in the AFF trial. This was largely due to difficulties in identifying patients while still in early-stage frozen shoulder and patient expectations regarding steroid injection. However, it was feasible to deliver the injection in line with trial protocol and to collect both patient and clinician assessed outcome data. Our interview findings indicated positive experiences from participants who took part in the trial.

For peer review only - http://bmjopen.bmj.com/site/about/guidelines.xhtml

Acknowledgments: This study is coordinated by the UKCRC registered Oxford Clinical Trials Research Unit (OCTRU) at the University of Oxford. It followed their Standard Operating Procedures, ensuring compliance with the principles of Good Clinical Practice and the Declaration of Helsinki and any appliable regulatory requirements. Special thanks go Nicola Kenealy as trial manager and Sue Dutton as co-applicant (now retired) and members of the Trial Oversight Committee: Ben Fisher (chair; University of Birmingham), Jim Wiggle (Patient Representative), Rebecca Evans (University of Bristol), Mike Thomas (Heatherwood and Wexham Park NHS Hospitals Trust), Anju Jaggi (Royal National Orthopaedic Hospital NHS Trust).

Collaborators: The Anti-Freaze-F study collaborating principal investigators were Vincent Gallagher (East Sussex Healthcare NHS Trust), Neil Smith (Sandwell and West Birmingham Hospital NHS Trust), Alun Yewlett (United Lincolnshire Hospitals NHS Trust), Paul Barker (University Hospitals Birmingham).

Contributors: SH and JN are co-chief investigators and co-lead applicants. AR, MF, JP and SL are co-applicants on the grant awarded by the National Institute of Health Research - Research for Patient Benefit Programme (Project reference: 201031) and were involved in design and conduct of the study, and interpretation of data. CS led the qualitative study, FE and SM conducted the statistical analysis and AE and EAF provided trial management expertise. SH, JN and CS led on writing the manuscript. All authors read, commented and approved the final version.

Funding: This report is independent research funded by the National Institute of Health Research - Research for Patient Benefit Programme (NIHR: 201031) The views expressed in this publication are those of the author(s) and not necessarily those of the NIHR or the Department of Health and Social Care. 180 Life Sciences provided funding for the purchase of adalimumab, the investigational medicinal product used in this trial.

Disclaimer: The views expressed are those of the author(s) and not necessarily those of the NHS, the NIHR or the Department of Health.

Competing interests: Professor Sir Marc Feldman and Professor Jagdeep Nanchahal are co-founders and hold equity in 180 Life Sciences, the company who funded the purchase of adalimumab, the investigational medicinal product used in this trial.

For peer review only

Ethics approval: Health Research Authority North East - Newcastle & North Tyneside 1 (REC 21/NE/0214).

Data sharing statement: Summary results data will be available on the trial registration databases within 12 months of the end of the trial. Requests for data (anonymised trial participant level data) will be provided at the end of the trial to external researchers who provide a methodologically sound proposal to the trial team (and who will be required to sign a data sharing access agreement with the Sponsor) and in accordance with the NIHR guidance. Participant consent for this was included in the informed consent form for the study.

For peer review only

REFERENCES

1. Dawson, J., Shepperd, S., Carr, A., *An overview of factors relevant to undertaking research and reviews on the effectiveness of treatment for frozen shoulder.* Shoulder and Elbow,, 2010. **2**: p. 232-237.
2. Walker-Bone, K., et al., *Prevalence and impact of musculoskeletal disorders of the upper limb in the general population.* Arthritis Rheum, 2004. **51**(4): p. 642-51.
3. Hand, C., et al., *Long-term outcome of frozen shoulder.* J Shoulder Elbow Surg, 2008. **17**(2): p. 231-6.
4. Rangan, A., N. Hanchard, and C. McDaid, *What is the most effective treatment for frozen shoulder?* Bmj, 2016. **354**: p. i4162.
5. Hanchard, N.C., et al., *Evidence-based clinical guidelines for the diagnosis, assessment and physiotherapy management of contracted (frozen) shoulder: quick reference summary.* Physiotherapy, 2012. **98**(2): p. 117-20.
6. Ryan, V., et al., *The pathophysiology associated with primary (idiopathic) frozen shoulder: A systematic review.* BMC Musculoskelet Disord, 2016. **17**(1): p. 340.
7. Buchbinder, R., et al., *Oral steroids for adhesive capsulitis.* Cochrane Database Syst Rev, 2006(4): p. Cd006189.
8. Buchbinder, R., S. Green, and J.M. Youd, *Corticosteroid injections for shoulder pain.* Cochrane Database Syst Rev, 2003(1): p. Cd004016.
9. Wang, W., et al., *Effectiveness of corticosteroid injections in adhesive capsulitis of shoulder: A meta-analysis.* Medicine (Baltimore), 2017. **96**(28): p. e7529.
10. Page, M.J., et al., *Electrotherapy modalities for adhesive capsulitis (frozen shoulder).* Cochrane Database Syst Rev, 2014(10): p. Cd011324.
11. Page, M.J., et al., *Manual therapy and exercise for adhesive capsulitis (frozen shoulder).* Cochrane Database Syst Rev, 2014(8): p. Cd011275.
12. Rangan, A., et al., *Management of adults with primary frozen shoulder in secondary care (UK FROST): a multicentre, pragmatic, three-arm, superiority randomised clinical trial.* Lancet, 2020. **396**(10256): p. 977-989.
13. Smith, S.P., V.S. Devaraj, and T.D. Bunker, *The association between frozen shoulder and Dupuytren's disease.* J Shoulder Elbow Surg, 2001. **10**(2): p. 149-51.
14. Larsen, S., et al., *Genetic and environmental influences in Dupuytren's disease: a study of 30,330 Danish twin pairs.* J Hand Surg Eur Vol, 2015. **40**(2): p. 171-6.
15. Riesmeijer, S.A., et al., *A genome-wide association meta-analysis implicates Hedgehog and Notch signaling in Dupuytren's disease.* Nat Commun, 2024. **15**(1): p. 199.
16. Bunker, T.D. and P.P. Anthony, *The pathology of frozen shoulder. A Dupuytren-like disease.* J Bone Joint Surg Br, 1995. **77**(5): p. 677-83.
17. Hand, G.C., et al., *The pathology of frozen shoulder.* J Bone Joint Surg Br, 2007. **89**(7): p. 928-32.
18. Bunker, T.D., et al., *Expression of growth factors, cytokines and matrix metalloproteinases in frozen shoulder.* J Bone Joint Surg Br, 2000. **82**(5): p. 768-73.
19. Lho, Y.M., et al., *Inflammatory cytokines are overexpressed in the subacromial bursa of frozen shoulder.* J Shoulder Elbow Surg, 2013. **22**(5): p. 666-72.
20. Nanchahal, J., et al., *Anti-Tumour Necrosis Factor Therapy for Dupuytren's Disease: A Randomised Dose Response Proof of Concept Phase 2a Clinical Trial.* EBioMedicine, 2018. **33**: p. 282-288.
21. Nanchahal, J., et al., *Anti-tumour necrosis factor therapy for early-stage Dupuytren's disease (RIDD): a phase 2b, randomised, double-blind, placebo-controlled trial.* Lancet Rheumatol, 2022. **4**(6): p. E407-e416.
22. British Elbow and Shoulder Society, B.O.A., Royal College of Surgeons, *Commissioning guide: subacromial shoulder pain*. 2014.

For peer review only - http://bmjopen.bmj.com/site/about/guidelines.xhtml

23. Hopewell, S., et al., *Anti-TNF (adalimumab) injection for the treatment of adults with frozen shoulder during the pain predominant stage protocol for a multi-centre, randomised, double blind, parallel group, feasibility trial [version 2; peer review: 2 approved].* . NIHR Open Res, 2023. **2:28**.
24. Sun, Y., et al., *The Effect of Corticosteroid Injection Into Rotator Interval for Early Frozen Shoulder: A Randomized Controlled Trial.* Am J Sports Med, 2018. **46**(3): p. 663-670.
25. Roach, K.E., et al., *Development of a shoulder pain and disability index.* Arthritis Care Res, 1991. **4**(4): p. 143-9.
26. Williams, J.W., Jr., D.R. Holleman, Jr., and D.L. Simel, *Measuring shoulder function with the Shoulder Pain and Disability Index.* J Rheumatol, 1995. **22**(4): p. 727-32.
27. Mintken, P.E., et al., *Psychometric properties of the Fear-Avoidance Beliefs Questionnaire and Tampa Scale of Kinesiophobia in patients with shoulder pain.* Arch Phys Med Rehabil, 2010. **91**(7): p. 1128-36.
28. Nicholas, M.K., B.E. McGuire, and A. Asghari, *A 2-item short form of the Pain Self-efficacy Questionnaire: development and psychometric evaluation of PSEQ-2.* J Pain, 2015. **16**(2): p. 153-63.
29. Bastien, C.H., A. Vallieres, and C.M. Morin, *Validation of the Insomnia Severity Index as an outcome measure for insomnia research.* Sleep Med, 2001. **2**(4): p. 297-307.
30. Kamper, S.J., C.G. Maher, and G. Mackay, *Global rating of change scales: a review of strengths and weaknesses and considerations for design.* J Man Manip Ther, 2009. **17**(3): p. 163-70.
31. Teare, M.D., et al., *Sample size requirements to estimate key design parameters from external pilot randomised controlled trials: a simulation study.* Trials, 2014. **15**: p. 264.
32. Braun, V.C., V., *Using thematic analysis in psychology.* Qualitative Research in Psychology, 2006. **3**(2): p. 77-101.
33. Limb, M., *Long NHS waits and rising living costs are harming people's health, show ONS data.* Bmj, 2023. **380**: p. 479.
34. Maund, E., et al., *Management of frozen shoulder: a systematic review and cost-effectiveness analysis.* Health Technol Assess, 2012. **16**(11): p. 1-264.
35. US Food and Drug Adminstration: www.accessdata.fda.gov/drugsatfda_docs/label/2018/125057s406lbl.pdf [accessed 05 July 2023].
36. *Office of National Statistics. Ethnicity https://www.ons.gov.uk/peoplepopulationandcommunity/culturalidentity/ethnicity [accessed 05 July 2023].*
37. Hopewell, S., et al., *Progressive exercise compared with best practice advice, with or without corticosteroid injection, for the treatment of patients with rotator cuff disorders (GRASP): a multicentre, pragmatic, 2 × 2 factorial, randomised controlled trial.* Lancet, 2021. **398**(10298): p. 416-428.

For peer review only - http://bmjopen.bmj.com/site/about/guidelines.xhtml

Figure 1: Participant flow diagram

[insert figure 1]

For peer review only

For peer review only - http://bmjopen.bmj.com/site/about/guidelines.xhtml

Table 1: Baseline characteristics by intervention group

	Adalimumab (n=4)	Placebo (n=5)	Total (n=9)
**Age**[1]	57.5 (5.9)	52.8(5.0)	54.9 (5.6)
**Sex**[2]			
Male	3 (75.0%)	3 (60.0%)	6 (66.7%)
Female	1 (25.0%)	2 (40.0%)	3 (33.3%)
**Ethnicity**[2]			
White British	4 (100.0%)	5 (100.0%)	9 (100.0%)
**Handedness**[2]			
Left	2 (50.0%)	1 (20.0%)	3 (33.3%)
Right	2 (50.0%)	4 (80.0%)	6 (66.7%)
**Duration of symptoms** (weeks)[1]	19.5 (13.1)	12.2 (2.3)	15.4 (9.0)
**Affected shoulder**[2]			
Left	3 (75.0%)	2 (40.0%)	5 (55.6%)
Right	1 (25.0%)	3 (60.0%)	4 (44.4%)
**BMI (kg/m²)**[1]	29.5 (5.8)	30.3 (5.3)	30.0 (5.2)
**Smoking status**[2]			
Never smoked	4 (100.0%)	3 (60.0%)	7 (77.8%)
Former smoker	0	2 (40.0%)	2 (22.2%)
If ever smoked, number of cigarettes/day[1]	0	12 (11.3)	12 (11.3)
**Dupuytren's disease**[2]			
No	4 (100.0%)	4 (80.0%)	8 (88.9%)
Yes	0	1 (20.0%)	1 (11.1%)
**Diabetes**[2]			
Type 1	0	1 (20.0%)	1 (11.1%)
Type 2	1 (25.0%)	2 (40.0%)	3 (33.3%)
No	3 (75.0%)	2 (40.0%)	5 (55.6%)

[1] *Summaries are mean (SD)*

[2] *Summaries are n (%)*

For peer review only

Table 2: Injection delivery

	Adalimumab (n=4)	Placebo (n=5)	Overall (n=9)
**First injection given**[1]	4 (100%)	5 (100%)	9 (100%)
Time from randomisation to first injection (days)[2]	12.3 (4.8)	7.2 (4.1)	9.4 (4.9)
Injection administered?[1]			
Yes – fully	4 (100%)	5 (100%)	9 (100%)
**Second injection given**[1]	4 (100%)	5 (100%)	9 (100%)
Time from first to second injection (days)[2]	18.5 (3.3)	20.8 (5.9)	19.8 (4.8)
Injection adminstered?[1]			
Yes – fully	4 (100%)	5 (100%)	9 (100%)

[1] *Summaries are n (%)*

[2] *Summaries are mean (SD)*

For peer review only - http://bmjopen.bmj.com/site/about/guidelines.xhtml

Table 3: Themes and participant quotes from qualitative interviews

Themes	Narrative quotes
Experiences with pain-predominant phase of frozen shoulder	• *'I always put childbirth as a 10, so I always come back from that really, so I would say it was kind of like an eight to nine* [Adalimumab]<br>• *'It affects when I pretty much … every day, whatever I'm doing'* [Placebo]<br>• *' it just doesn't help me sleeping. I'm getting about three hours and max four hours at nights…'* [Placebo]
Perceptions about participation in the trial	• *'…if it's gonna help me and help other people then, yeah, it's something I'd do'* [Placebo]<br>• *'My first thought was yes, I was very happy to, primarily because the current treatment hadn't been particularly successful and I was in still quite a bit of pain and struggling to sleep very well and I thought the fact that another option was being made available, I was more than comfortable to participate'* [Adalimumab]<br>• *'my expectation really was that if I did get the drug it was likely that I would see some kind of improvement'* [Adalimumab]<br>• *'I hoped that I got the drug and not the placebo and it helped'* [Adalimumab]<br>• *I accepted that that's now how drug trials work and, therefore, I knew there was a possibility that I may not be receiving the drug'* [Adalimumab]<br>• *'I mean initially I was concerned because if I was the placebo I felt that potentially I was gonna be, well maybe three months further down the line having had the placebo and no benefit. And then I'd have to go back to square one so I was slightly anxious about that. But I was reassured that it, you know, it wasn't stopping me then getting further treatment if I hadn't had any response to the injections. And I guess, this is a bit sceptical of me that waiting lists are what they are but I probably would have been waiting three months for a steroid injection anyway. So, I didn't think it as a disadvantage to do the trial really. If it worked, great, and if it didn't, then I'd-- like I say, I'd go back to the GP'* [Placebo]
Perceptions about trial processes	• *'I couldn't fault the information I was given to be honest, they were very good'* [Placebo]<br>• *'I just went in had the injection. It wasn't very pleasant. It was quite…quite painful. It did make me feel a little bit queasy at the time'* [Adalimumab]<br>• *'I was happy to do that because I understand that they have to gauge any improvement in my shoulder movements over time. So, yeah, I was happy to complete those movements'* [Placebo]<br>• *'I mean it all went well. I was seen on time, everything was ready. Everything went to plan'* [Adalimumab]<br>• *'I'm really delighted that I'm part of the trial now because I'm seeing the benefit of it already. So, I'm very … I'm thrilled. I feel very lucky… I mean I do feel-- I feel honoured and I feel like they've treated me so well'* [Placebo]
Perceptions about effects of adalimumab and placebo injections	<u>After first and second adalimumab injections</u><br>• *'I think there was a noticeable improvement from the first injection. Perhaps a slightly… bit of an extra improvement after the second one. But I think it was quite noticeable following that first injection'* [Adalimumab]<br>• *Perhaps a slightly… bit of an extra improvement after the second one. But I think it was quite noticeable following that first injection'.. I definitely have got significant reduction in the pain. I've got more what I would say is a tightness in the muscular areas now, so this almost feels as though where I was on the old regime – pre-trial'* [Adalimumab]<br><br><u>After first and second placebo injections</u><br>• *'once I had the first injection, my arm … the actual … the pain actually increased quite significantly in my shoulder for a good 7 to 10 days. And I was having to take strong painkillers to sort of manage that. It's actually settled down a bit now'* [Placebo]

For peer review only - http://bmjopen.bmj.com/site/about/guidelines.xhtml

	• *'…there wasn't any significant increase in pain after the second injection…I've not noticed any real improvement since I had the second injection…I still do get the discomfort that I've got in my shoulder'* [Placebo]

For peer review only

For peer review only - http://bmjopen.bmj.com/site/about/guidelines.xhtml

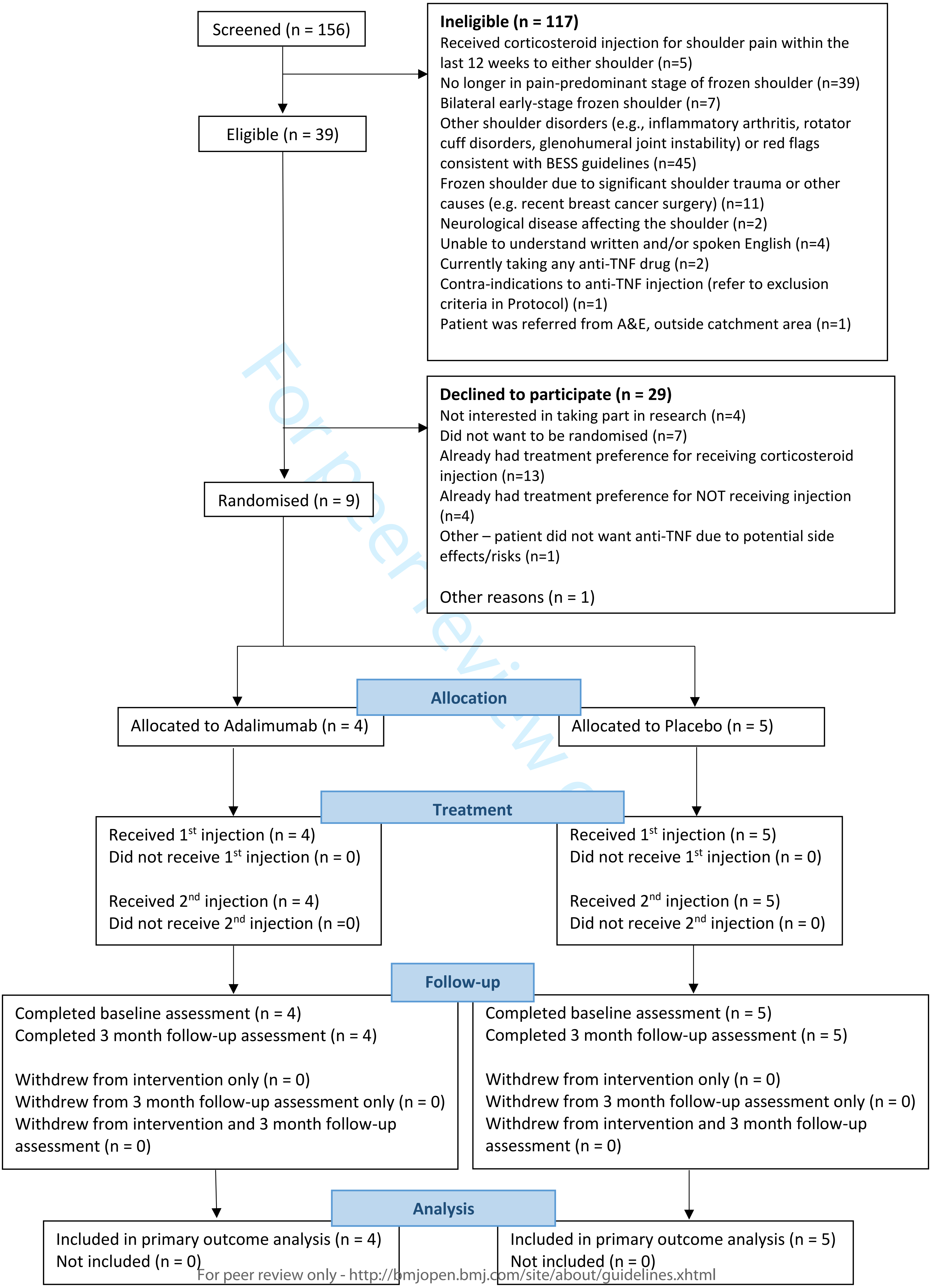

For peer review only

Supplementary materials (online only)

Figure S1: SPADI score: change from baseline and at 3 months

a) Total SPADI Scores at baseline and 3 months for each participant

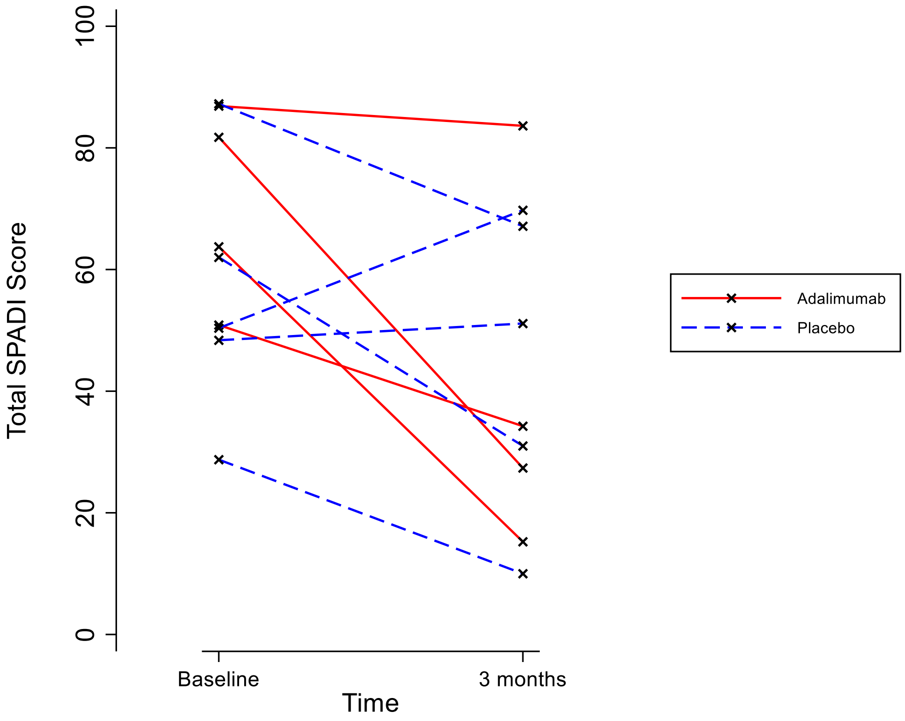

b) SPADI Pain Subscale Scores at baseline and 3 months for each participant

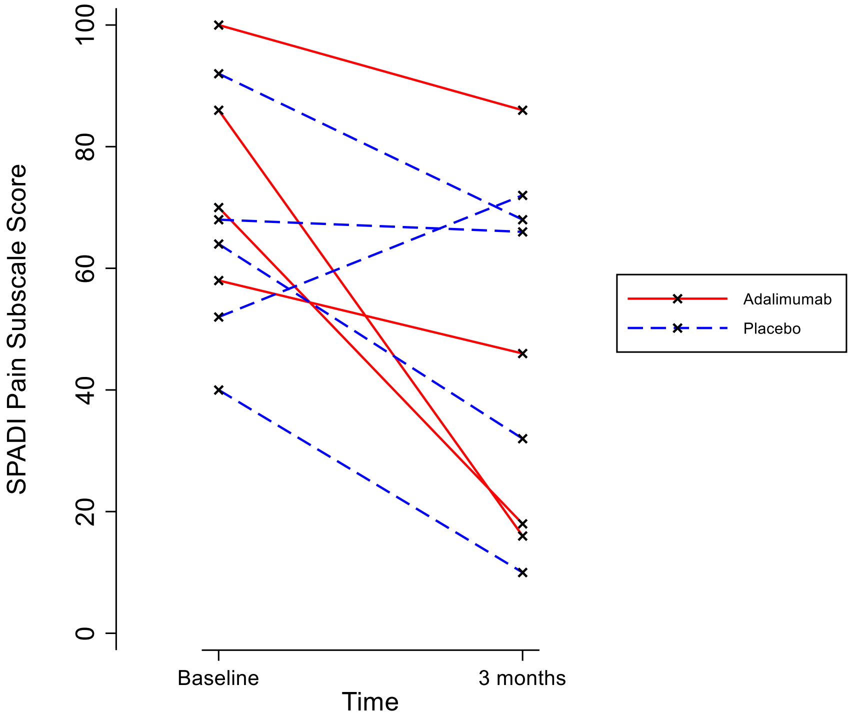

For peer review only - http://bmjopen.bmj.com/site/about/guidelines.xhtml

c) SPADI Function Subscale Scores at baseline and 3 months for each participant

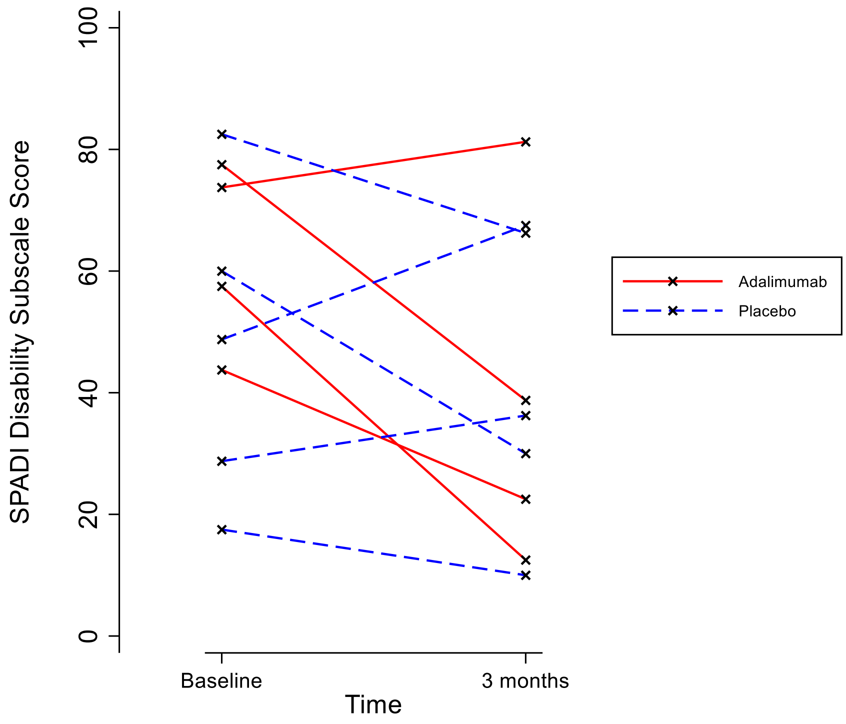

review only

Table S1: Secondary patient-reported outcomes

		Adalimumab[1] (n=4)	Placebo[1] (n=5)
SPADI Total score	Baseline	70.8 (16.6)	55.4 (21.5)
	3 months	40.1 (30.0)	45.8 (25.3)
SPADI pain (subscale)	Baseline	78.5 (18.4)	63.2 (19.5)
	3 months	41.5 (32.7)	49.6 (27.3)
SPADI disability (subscale)	Baseline	63.1 (15.6)	47.5 (25.7)
	3 months	38.8 (30.3)	42 (24.7)
FAB-Q	Baseline	17.3 (3.3)	17.4 (3.6)
	3 months	9.3 (6.2)	12.8 (5.9)
PSEQ – short-form	Baseline	9 (3.6)	9.2 (1.9)
	3 months	10.5 (1.3)	8.2 (2.8)
ISI	Baseline	17.8 (5.)	17.6 (4.0)
	3 months	9.8 (6.7)	13.2 (7.6)
Return to desired activities	Baseline	9 (4.5)	9.4 (2.7)
	3 months	5.5 (3.8)	8.4 (3.6)
Global impression of change	3 months	2.3 (2.2)	0 (3.0)

[1] *Summaries are mean (SD)*

SPADI: Shoulder Pain And Disability Index; FAB-Q: Fear-Avoidance Belief Questionnaire; PSEQ: Pain Self-Efficacy Questionnaire; ISI: Insomnia Severity Index

For peer review only

For peer review only - http://bmjopen.bmj.com/site/about/guidelines.xhtml

Table S2: Clinician-assessed range of shoulder motion

	Adalimumab[1] (n=4)	Placebo[1] (n=5)
**Flexion**		
Baseline	71.3 (26.3)	101.6 (24.7)
3 months	112.5 (54.8)	140 (25.7)
**Extension**		
Baseline	26.3 (18.0)	27.6 (9.5)
3 months	30.0 (8.2)	40.6 (9.8)
**Abduction**		
Baseline	50.0 (30.3)	69.0 (26.6)
3 months	105.0 (56.1)	115.0 (42.7)
**Internal rotation**		
Baseline	23.8 (18.9)	40.2 (40.8)
3 months	21.3 (17.0)	41 (37.8)
**External rotation**		
Baseline	22.5 (8.7)	15.0 (16.9)
3 months	28.8 (6.3)	55.0 (16.2)

[1] Summaries are mean (SD) degrees of shoulder motion

Table S3: Patient reported range of shoulder motion

	Adalimumab[1] (n=4)	Placebo[1] (n=5)
**Flexion**		
Baseline	102.3 (21.3)	112.4 (21.3)
3 months	117.3 (38.4)	145.6 (22.9)
**Extension**		
Baseline	26.3 (6.9)	23.7 (8.5)
3 months	23.5 (7.0)	36.5 (7.0)
**Abduction**		
Baseline	81.3 (35.4)	99.3 (27.0)
3 months	79.3 (45.7)	134.2 (52.5)
**Internal rotation**		
Baseline	18.5 (19.8)	29 (29.9)
3 months	51.0 (15.6)	72.3 (19.4)
**External rotation**		
Baseline	35.3 (15.7)	24.8 (14.5)
3 months	35.5 (9.8)	37.0 (24.0)

[1] Summaries are mean (SD) degrees of shoulder motion

For peer review only

For peer review only - http://bmjopen.bmj.com/site/about/guidelines.xhtml

RESEARCH METHODS AND REPORTING

Table 2 | CONSORT checklist of information to include when reporting a pilot trial

Section/topic and item No	Standard checklist item	Extension for pilot trials	Page No where item is reported
**Title and abstract**			
1a	Identification as a randomised trial in the title	Identification as a pilot or feasibility randomised trial in the title	1
1b	Structured summary of trial design, methods, results, and conclusions (for specific guidance see CONSORT for abstracts)	Structured summary of pilot trial design, methods, results, and conclusions (for specific guidance see CONSORT abstract extension for pilot trials)	2
**Introduction**			
Background and objectives:			
2a	Scientific background and explanation of rationale	Scientific background and explanation of rationale for future definitive trial, and reasons for randomised pilot trial	4-5
2b	Specific objectives or hypotheses	Specific objectives or research questions for pilot trial	4-5
**Methods**			
Trial design:			
3a	Description of trial design (such as parallel, factorial) including allocation ratio	Description of pilot trial design (such as parallel, factorial) including allocation ratio	5
3b	Important changes to methods after trial commencement (such as eligibility criteria), with reasons	Important changes to methods after pilot trial commencement (such as eligibility criteria), with reasons	N/A
Participants:			
4a	Eligibility criteria for participants		6
4b	Settings and locations where the data were collected		6
4c		How participants were identified and consented	6
Interventions:			
5	The interventions for each group with sufficient details to allow replication, including how and when they were actually administered		7
Outcomes:			
6a	Completely defined prespecified primary and secondary outcome measures, including how and when they were assessed	Completely defined prespecified assessments or measurements to address each pilot trial objective specified in 2b, including how and when they were assessed	8
6b	Any changes to trial outcomes after the trial commenced, with reasons	Any changes to pilot trial assessments or measurements after the pilot trial commenced, with reasons	N/A
6c		If applicable, prespecified criteria used to judge whether, or how, to proceed with future definitive trial	8
Sample size:			
7a	How sample size was determined	Rationale for numbers in the pilot trial	8-9
7b	When applicable, explanation of any interim analyses and stopping guidelines		
Randomisation:			
Sequence generation:			
8a	Method used to generate the random allocation sequence		6
8b	Type of randomisation; details of any restriction (such as blocking and block size)	Type of randomisation(s); details of any restriction (such as blocking and block size)	6
Allocation concealment mechanism:			
9	Mechanism used to implement the random allocation sequence (such as sequentially numbered containers), describing any steps taken to conceal the sequence until interventions were assigned		6
Implementation:			
10	Who generated the random allocation sequence, enrolled participants, and assigned participants to interventions		6
Blinding:			
11a	If done, who was blinded after assignment to interventions (eg, participants, care providers, those assessing outcomes) and how		6-7
11b	If relevant, description of the similarity of interventions		6-7
Analytical methods:			
12a	Statistical methods used to compare groups for primary and secondary outcomes	Methods used to address each pilot trial objective whether qualitative or quantitative	9
12b	Methods for additional analyses, such as subgroup analyses and adjusted analyses	Not applicable	N/A

BMJ: first published as 10.1136/bmj.i5239 on 24 October 2016. Downloaded from http://www.bmj.com/ on 26 July 2023 at Bodleian Libraries of the University of Oxford. Protected by copyright.

Table 2 | CONSORT checklist of information to include when reporting a pilot trial

Section/topic and item No	Standard checklist item	Extension for pilot trials	Page No where item is reported
**Results**			
Participant flow (a diagram is strongly recommended):			
13a	For each group, the numbers of participants who were randomly assigned, received intended treatment, and were analysed for the primary outcome	For each group, the numbers of participants who were approached and/or assessed for eligibility, randomly assigned, received intended treatment, and were assessed for each objective	figure 1
13b	For each group, losses and exclusions after randomisation, together with reasons		Figure 1
Recruitment:			
14a	Dates defining the periods of recruitment and follow-up		9
14b	Why the trial ended or was stopped	Why the pilot trial ended or was stopped	9
Baseline data:			
15	A table showing baseline demographic and clinical characteristics for each group		Table 1, p20
Numbers analysed:			
16	For each group, number of participants (denominator) included in each analysis and whether the analysis was by original assigned groups	For each objective, number of participants (denominator) included in each analysis. If relevant, these numbers should be by randomised group	Table 2, p20 page 10
Outcomes and estimation:			
17a	For each primary and secondary outcome, results for each group, and the estimated effect size and its precision (such as 95% confidence interval)	For each objective, results including expressions of uncertainty (such as 95% confidence interval) for any estimates. If relevant, these results should be by randomised group	Table 2, p20 page 10
17b	For binary outcomes, presentation of both absolute and relative effect sizes is recommended	Not applicable	N/A.
Ancillary analyses:			
18	Results of any other analyses performed, including subgroup analyses and adjusted analyses, distinguishing prespecified from exploratory	Results of any other analyses performed that could be used to inform the future definitive trial	N/A
Harms:			
19	All important harms or unintended effects in each group (for specific guidance see CONSORT for harms)		
19a		If relevant, other important unintended consequences	N/A.
**Discussion**			
Limitations:			
20	Trial limitations, addressing sources of potential bias, imprecision, and, if relevant, multiplicity of analyses	Pilot trial limitations, addressing sources of potential bias and remaining uncertainty about feasibility	14
Generalisability:			
21	Generalisability (external validity, applicability) of the trial findings	Generalisability (applicability) of pilot trial methods and findings to future definitive trial and other studies	13-14
Interpretation:			
22	Interpretation consistent with results, balancing benefits and harms, and considering other relevant evidence	Interpretation consistent with pilot trial objectives and findings, balancing potential benefits and harms, and considering other relevant evidence	13-14
22a		Implications for progression from pilot to future definitive trial, including any proposed amendments	13-14
**Other information**			
Registration:			
23	Registration number and name of trial registry	Registration number for pilot trial and name of trial registry	2
Protocol:			
24	Where the full trial protocol can be accessed, if available	Where the pilot trial protocol can be accessed, if available	6
Funding:			
25	Sources of funding and other support (such as supply of drugs), role of funders		16
26		Ethical approval or approval by research review committee, confirmed with reference number	16

BMJ: first published as 10.1136/bmj.i5239 on 24 October 2016. Downloaded from http://www.bmj.com/ on 26 July 2023 at Bodleian Libraries of the University of Oxford. Protected by copyright.

 doi: 10.1136/bmj.i5239 | BMJ 2016;355:i5239 | the bmj

For peer review only - http://bmjopen.bmj.com/site/about/guidelines.xhtml
